# Midkine inhibition enhances anti-PD-1 immunotherapy in sorafenib-treated hepatocellular carcinoma via preventing immunosuppressive MDSCs infiltration

**DOI:** 10.1038/s41420-023-01392-3

**Published:** 2023-03-11

**Authors:** Lijuan Ding, Nanya Wang, Qiang Wang, Xia Fan, Yuning Xin, Shudong Wang

**Affiliations:** 1 Department of Radiation Oncology, the First Hospital of Jilin University, Changchun, 130021 China; 2 Cancer Center, the First Hospital of Jilin University, Changchun, 130021 China; 3 Department of Cardiology, the First Hospital of Jilin University, Changchun, 130021 China

**Keywords:** Cancer microenvironment, Cancer therapy

## Abstract

Sorafenib, a multiple-target tyrosine kinase inhibitor, is the standard of care for patients with advanced hepatocellular carcinoma (HCC), but provides limited benefits. Emerging evidences suggest that prolonged sorafenib treatment induces an immunosuppressive HCC microenvironment, but the underling mechanism is undetermined. In the present study, the potential function of midkine, a heparin-binding growth factor/cytokine, was evaluated in sorafenib-treated HCC tumors. Infiltrating immune cells of orthotopic HCC tumors were measured by flow cytometry. Differentially expressed genes in sorafenib-treated HCC tumors were evaluated by transcriptome RNA sequencing. The potential function of midkine were evaluated by western blot, T cell suppression assay, immunohistochemistry (IHC) staining and tumor xenograft model. We found that sorafenib treatment increased intratumoral hypoxia and altered HCC microenvironment towards an immune-resistant state in orthotopic HCC tumors. Sorafenib treatment promoted midkine expression and secretion by HCC cells. Moreover, forced midkine expression stimulated immunosuppressive myeloid-derived suppressor cells (MDSCs) accumulation in HCC microenvironment, while knockdown of midkine exhibited opposite effects. Furthermore, midkine overexpression promoted CD11b^+^CD33^+^HLA-DR^−^ MDSCs expansion from human PBMCs, while midkine depletion suppressed this effect. PD-1 blockade showed no obvious inhibition on tumor growth of sorafenib-treated HCC tumors, but the inhibitory effect was greatly enhanced by midkine knockdown. Besides, midkine overexpression promoted multiple pathways activation and IL-10 production by MDSCs. Our data elucidated a novel role of midkine in the immunosuppressive microenvironment of sorafenib-treated HCC tumors. Mikdine might be a potential target for the combination of anti-PD-1 immunotherapy in HCC patients.

## Introduction

Hepatocellular carcinoma (HCC) ranks the sixth most common cancer and second leading cause of cancer-related death worldwide [1]. In the last two decades, the incidence and mortality rates of HCC are increasing in most parts of the world, especially eastern Asia and Africa [2]. Patients with HCC have a really poor prognosis, largely due to late diagnosis and high recurrence rate. The majority of HCCs are diagnosed at intermediate or advanced stages, with a 5-year survival rate as low as 16% [3]. Patients with HCC are refractory to almost all conventional chemotherapeutic drugs. Systemic therapy for advanced HCC consists only of antiangiogenic tyrosine kinase inhibitors until recently. Sorafenib, a multiple-target tyrosine kinase inhibitor with antiangiogenic and antiproliferation effects, is approved by the Food and Drug Administration (FDA) as the first-line chemotherapeutic drug for advanced HCC. In two large phase III trials, sorafenib is proved to be effective in advanced HCC patients [4, 5]. However, less than 30% of HCC patients can benefit from sorafenib treatment, and the median survival is extended only about 2.5 months [5]. Besides, these patients usually become resistant to sorafenib within 6 months [6]. Another chemotherapeutic drug lenvatinib is not superior to sorafenib when used in the first-line setting [7]. Thus, there is urging needing to develop novel therapeutic strategies.

HCC is typically caused by chronic inflammation of a liver diseases [8]. Therefore, there is a strong rationale for using of immunotherapy. Immune checkpoints include co-inhibitory receptors such as programmed cell death-1 (PD-1) and its ligand programmed death ligand 1 (PD-L1). PD-1 is expressed by an extensive number of immune cells such as activated T cells and natural killer (NK) cells, while PD-L1 is expressed on tumor cells, stromal cells and myeloid cells [9]. In general, antiangiogenic therapy with sorafenib, lenvatinib or VEGF antibodies remains the fundamental treatment for advanced HCC, whereas immunotherapy with checkpoint inhibitors becomes increasing important [10]. Recently, immunotherapy with checkpoint inhibitors has been tested in HCC patients and shows strong anti-tumor effect in a subset of patients [11, 12]. The combination of PD-1/PD-L1 and vascular endothelial growth factor (VEGF) antibodies results in a better survival than sorafenib, which makes it as a new first line therapy [13]. Despite these improvements, only one in four patients respond to these immunotherapies, and the majority of HCC patients do not respond due to unknown reasons. One aspect is that prolonged antiangiogenic therapy increases intratumoral hypoxia, which facilitates tumor recurrence and fosters an immunosuppressive microenvironment [14, 15]. Since the efficiency of immune checkpoint inhibitors is greatly influenced by immunosuppressive tumor microenvironment, it is necessary to elucidate the underlying mechanism.

Midkine (MDK) is a heparin-binding growth factor/cytokine that exhibits multiple functions and implicates in various physiological process [16]. Midkine is rarely expressed by normal tissues, but significantly upregulated in inflammatory diseases and human malignant tumors [17]. Upregulation of midkine can promote growth, survival, metastasis, and angiogenesis of cancer cells [16]. Moreover, a number of immune cells are influenced by midkine, including macrophage [18], polymorphonuclear (PMN) [19], B cells [20] and T cells [21]. In addition, midkine plays a vital role in maintaining immunosuppressive tumor microenvironment of melanoma and gallbladder cancer [22, 23]. Midkine is upregulated in HCC patients and associated with poor prognosis [24]. However, the potential role of midkine in HCC microenvironment is undetermined. Myeloid-derived suppressor cells (MDSCs) are immature marrow-derived cell populations with potent immunosuppressive activity. They are mainly consisted of granulocytic/polymorphonuclear MDSCs (PMN-MDSCs) and monocytic MDSCs (M-MDSCs), which are similar to neutrophils and monocytes, respectively. In the present study, orthotopic HCC models were constructed to evaluate the potential role of midkine in sorafenib-induced immunosuppressive microenvironment. We found that midkine overexpression stimulated MDSCs infiltrating into HCC tumors, while knockdown of midkine exhibited opposite effects. In addition, midkine inhibition enhanced the inhibitory effects of anti-PD-1 immunotherapy in sorafenib-treated HCC tumors. Our results elucidated a novel role of midkine in HCC microenvironment, and midkine might be a potential target for HCC treatment.

## Results

### Sorafenib treatment increases intratumoral hypoxia and alters HCC microenvironment towards an immune-resistant state in mouse models

**Fig. 1:**
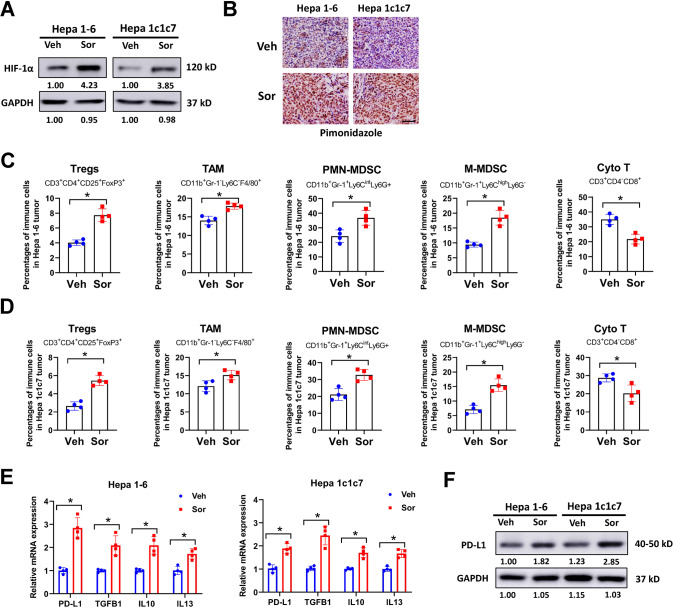
Sorafenib treatment increases intratumoral hypoxia and alters HCC microenvironment towards an immune-resistant state in mouse models. **A**, **B** orthotopic Hepa 1-6 and Hepa 1c1c7 tumors were treated with sorafenib or vehicle control for three weeks, then collected protein lysates for western blot (**A**) or tissue samples for Pimonidazole staining (**B**). **C**, **D** infiltrating immune cells in orthotopic Hepa 1-6 and Hepa 1c1c7 tumors treated with sorafenib or vehicle control were evaluated by flow cytometry. CD3^+^CD4^+^CD25^+^FoxP3^+^ Treg, CD11b^+^Gr-1^−^Ly6C^−^F4/80^+^ TAM, CD11b^+^Gr-1^+^Ly6C^int^Ly6G^+^ PMN-MDSC, CD11b^+^Gr-1^+^Ly6C^high^Ly6G^-^ M-MDSC and CD3^+^CD4^-^CD8^+^ Cyto T cells were evaluated. **E** relative expression of PD-L1, TGFB1, IL10, and IL13 in orthotopic Hepa 1-6 and Hepa 1c1c7 tumors was evaluated by qRT-PCR. **F** protein expression of PD-L1 in orthotopic Hepa 1-6 and Hepa 1c1c7 tumors was evaluated by western blot. All assays were done with at least three repeats. Data were shown as mean ± s.d., *P < 0.05.

To determine whether sorafenib-induced intratumoral hypoxia may foster an immunosuppressive microenvironment, orthotopic HCC models were constructed using murine HCC cell lines Hepa 1-6 and Hepa 1c1c7 in C57BL/6 mice. In our study, sorafenib treatment significantly increased the protein expression of HIF1-α in orthotopic Hepa 1-6 and Hepa 1c1c7 tumors (Fig. 1A). Pimonidazole is a hypoxia-specific marker. Sorafenib treatment evidently increased the number of pimonidazole positive cells in orthotopic Hepa 1-6 and Hepa 1c1c7 tumors (Fig. 1B). In HCC patients, sorafenib treatment significantly increased HIF1-α expression and reduced vessel density in tumor samples (Supplementary Fig. 1A–C). Moreover, HIF1-α dependent genes such as VEGF, GLUT-1, CA-9, CXCR4, and MDR1 were upregulated in sorafenib-treated patients (Supplementary Fig. 1D). These results indicated that sorafenib increased intratumoral hypoxia in orthotopic HCC models and patient samples. To evaluate the immune microenvironment of orthotopic HCC tumors, infiltrating immune cells were evaluated by flow cytometry. The gating strategy and representative plots of infiltrating immune cell populations were depicted in Supplementary Fig. 2. The percentages of regulator T cells (Treg, CD3^+^CD4^+^CD25^+^FoxP3^+^), tumor-associated macrophages (TAM, CD11b^+^Gr-1^−^Ly6C^-^F4/80^+^), polymorphonuclear myeloid-derived suppressor cells (PMN-MDSC, CD11b^+^Gr-1^+^Ly6C^int^Ly6G^+^) and monocytic myeloid-derived suppressor cells (M-MDSC, CD11b^+^Gr-1^+^Ly6C^high^Ly6G^-^) were obviously increased, while cytotoxic T cells (Cyto T, CD3^+^CD4^−^CD8^+^) were apparently decreased in sorafenib-treated HCC tumors (Fig. 1C, D). PD-L1, TGFB1, IL10, and IL13 are primary effectors for immunosuppressive tumor microenvironment [25, 26]. These genes were significantly upregulated in sorafenib-treated HCC tumors (Fig. 1E). The protein expression of PD-L1 was also increased by sorafenib (Fig. 1F). Our results indicated that sorafenib treatment altered HCC microenvironment toward an immune-resistant state in mouse models.

### Sorafenib treatment promotes midkine expression and secretion by HCC cells

**Fig. 2:**
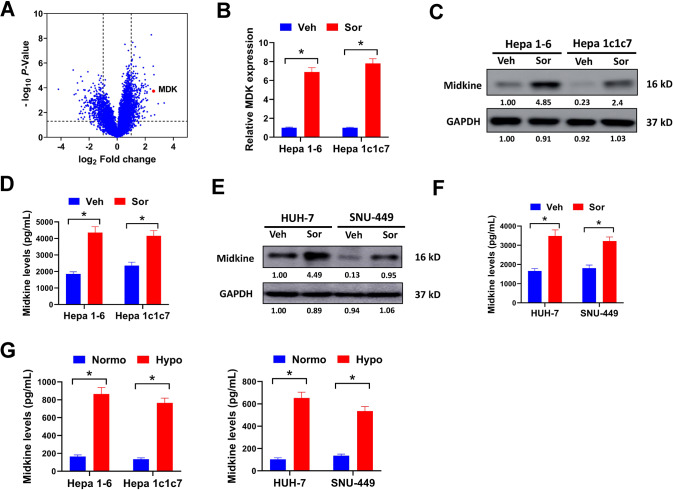
Sorafenib treatment promotes midkine expression and secretion by HCC cells. **A** differentially expressed genes in orthotopic Hepa 1-6 tumors treated with sorafenib (Sor) or vehicle control (Veh) were depicted in volcano map. **B**, **C** midkine expression in orthotopic Hepa 1-6 and Hepa 1c1c7 tumors treated with sorafenib or vehicle control was evaluated by qRT-PCR (**B**) and western blot (**C**). **D** secreted mikdine in serum samples of orthotopic Hepa 1-6 and Hepa 1c1c7 tumors treated with sorafenib or vehicle control was measured by ELISA assay. **E**, **F** midkine expression in orthotopic HUH-7 and SNU-449 tumors treated with sorafenib or vehicle control was evaluated by qRT-PCR (**E**) and western blot (**F**). **G** secreted mikdine in serum samples of orthotopic HUH-7 and SNU-449 tumors treated with sorafenib or vehicle control was measured by ELISA assay. All assays were done with at least three repeats. Data were shown as mean ± s.d., *P < 0.05.

To search for potential genes involved in sorafenib-induced immunosuppressive microenvironment, orthotopic Hepa 1-6 tumors treated with sorafenib or vehicle control were subjected to transcriptome RNA sequencing. In our study, 71 genes were significantly upregulated while 131 genes were downregulated in sorafenib-treated tumors compared with vehicle-treated tumors (Fig. 2A, Supplementary Table 1). Among them, midkine ranked the top ten upregulated genes (Fig. 2A, Supplementary Table 1). Midkine plays an important role in maintaining immunosuppressive microenvironment of melanoma and gallbladder cancer [22, 23]. Thus, we speculated that the upregulation of midkine might contribute to the immune-resistant state caused by sorafenib treatment in HCC tumors. In our study, midkine expression in pan-cancer was evaluated by TIMER database. We found that midkine was significantly upregulated in a variety of cancers, including HCC (Supplementary Fig. 3A). Data from TCGA and GEO database (GSE39791 and GSE112790) also suggested that midkine was overexpressed in HCC tumor samples (Supplementary Fig. 3B and C). Besides, high midkine expression was positively correlated with advanced tumor stages and poor overall-survival of HCC patients (Supplementary Fig. 3D and E). In our study, midkine expression was increased by sorafenib in orthotopic HCC tumors (Figs. 2B and 2C). Midkine is a secreted protein. Sorafenib treatment apparently increased the level of secreted midkine in serum samples of mice bearing HCC tumors (Fig. 2D). This was also validated in human HCC cell lines HUH-7 and SNU-449. Sorafenib treatment evidently augmented midkine level in tumor tissues and serum samples of Balb/c nude mice bearing orthotopic HUH-7 or SNU-449 tumors (Fig. 2E, F). The promoter region of midkine has a hypoxia responsive element, thus hypoxia may induce midkine expression via binding with HIF-1α [27]. This was tested in HCC cells. Secreted midkine was significantly elevated in culture medium of both murine and human HCC cells under hypoxia condition (1% O_2_) compared with normoxia condition (20% O_2_) (Fig. 2G). The above results indicated that sorafenib treatment promoted midkine expression and secretion by HCC cells.

### Forced midkine expression stimulates immunosuppressive MDSCs accumulation in HCC tumor microenvironment

**Fig. 3:**
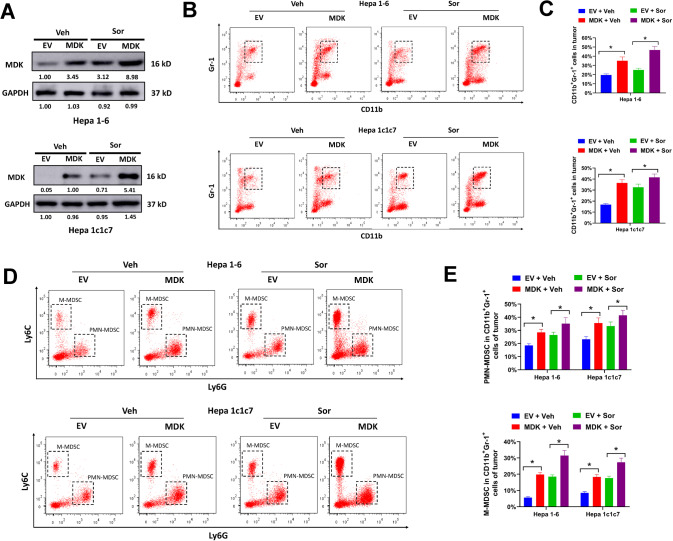
Hepatocellular midkine overexpression stimulates MDSCs accumulation in tumor microenvironment. Midkine-overexpression or EV-transduced Hepa 1-6 and Hepa 1c1c7 cells were orthotopically implanted into the liver of C57BL/6 mice, then treated with sorafenib (Sor) or vehicle control (Veh) for three weeks. **A** protein expression of midkine was validated by western blot. **B**, **C** infiltrating CD11b^+^Gr-1^+^ cells were evaluated by flow cytometry. **D**, **E** Ly6C^int^Ly6G^+^ PMN-MDSCs and Ly6C^high^Ly6G^-^ M-MDSCs in infiltrating CD11b^+^Gr-1^+^ subset were evaluated by flow cytometry. All assays were done with at least three repeats. Data were shown as mean ± s.d., *P < 0.05.

To elucidate the potential effects of midkine in HCC microenvironment, the correlation between midkine and tumor immune infiltration was evaluated. The 22 tumor-infiltrating immune cells in HCC tissues were estimated by CIBERSORT algorithm. HCC patients with high midkine expression showed enriched scores for Treg (Supplementary Fig. 4). Besides, high midkine expression was positively associated with immune checkpoint molecules such as CTLA4, HAVCR2, LAG3, PDCD1, and TIGIT (Supplementary Fig. 5A). TISIDB was also used to investigate the correlation of midkine with infiltrating immune cells. High midkine expression was positively correlated with MDSCs abundance, but not monocytes or neutrophils (Supplementary Fig. 5B). The data from TIMER demonstrated that immune makers of MDSCs (CD33, ITGAM and FUT4) were significantly associated with midkine expression (Supplementary Fig. 5C). These results suggested that midkine might involve in tumor infiltrating of MDSCs. To validate this, midkine was overexpressed by transducing with midikine expression lentivirial particles (Fig. 3A). CD11b^+^ and Gr-1^+^ are myeloid differentiation markers for mouse MDSCs. Forced midkine expression apparently increased the percentages of infiltrating CD11b^+^Gr-1^+^ cells in orthotopic Hepa 1-6 and Hepa 1c1c7 tumors, and this effect was enforced by sorafenib treatment (Fig. 3B, C). There are two main subsets of mouse MDSCs: PMN-MDSCs (CD11b^+^Gr-1^+^Ly6C^int^Ly6G^+^) and M-MDSCs (CD11b^+^Gr-1^+^Ly6C^high^Ly6G^−^). Midkine overexpression significantly increased the percentages of both PMN-MDSCs and M-MDSCs in orthotopic Hepa 1-6 and Hepa 1c1c7 tumors with/without sorafenib treatment (Fig. 3D, E).

**Fig. 4:**
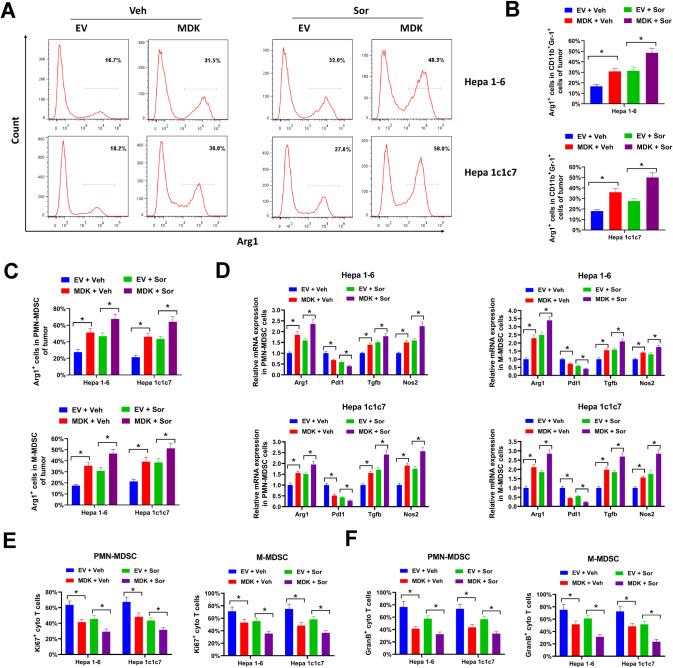
Hepatocellular midkine overexpression induces T cell-suppressive MDSC accumulation in tumor microenvironment. Midkine-overexpression or EV-transduced Hepa 1-6 and Hepa 1c1c7 cells were orthotopically implanted into the liver of C57BL/6 mice, then treated with sorafenib (Sor) or vehicle control (Veh) for three weeks. A-C, Arg1^+^ cells in infiltrating CD11b^+^Gr-1^+^ subset (**A**–**B**) and MDSCs subset (**C**) of orthotopic Hepa 1-6 and Hepa 1c1c7 tumors were evaluated by flow cytometry. **D** Relative expression of Arg1, Pdl1, Tgfb and Nos2 in infiltrating PMN-MDSCs and M-MDSCs subsets of orthotopic Hepa 1-6 and Hepa 1c1c7 tumors was evaluated by qRT-PCR. **E**, **F** Infiltrating PMN-MDSCs and M-MDSCs of orthotopic Hepa 1-6 and Hepa 1c1c7 tumors were used for T-cell suppression assay. Ki67^+^ (**E**) and Granzyme B^+^ (**F**) cytotoxic T cells were evaluated by flow cytometry. All assays were done with at least three repeats. Data were shown as mean ± s.d., *P < 0.05.

Compared with monocytes or neutrophils, mouse MDSCs are characterized by high level of arginase 1 (Arg1) [28]. Of note, Arg1 is vital for the immunosuppression function of MDSCs. In our study, midkine overexpression dramatically increased the number of Arg1^+^ cells in CD11b^+^Gr-1^+^ subset of orthotopic HCC tumors with/without sorafenib treatment (Fig. 4A, B). Moreover, the percentages of Arg1^+^ cells in PMN-MDSCs and M-MDSCs subsets were also increased by ectopic midkine expression (Fig. 4C). Arg1, Pdl1, Tgfb and Nos2 are primary effectors for the immunosuppression function of MDSCs. Forced midkine expression increased the expression of Arg1, Tgfb and Nos2, and decreased the expression of Pdl1 in sorted PMN-MDSCs and M-MDSCs (Fig. 4D). The immunosuppression activity of MDSCs in midkine-overexpressing HCC tumors was further evaluated by T-cell suppression assay. CD3^+^CD8^+^ cytotoxic T cells were stimulated with CD3e and CD28 antibodies and co-cultured with MDSCs sorted from midkine-overexpressing HCC tumors or EV-transduced tumors for three days. Cytotoxic T cells co-cultured with MDSCs sorted from midkine-overexpressing HCC tumors showed reduced Ki67 and Granzyme B expression compared with T cells co-cultured with MDSCs derived from EV-transduced tumors, indicating that the proliferation and activation of T cells were repressed (Fig. 4E, F). Moreover, MDSCs derived from sorafenib-treated tumors showed enhanced inhibition on T cell proliferation and activation, especially those from midkine-overexpressing tumors (Fig. 4E, F). These results indicated that MDSCs derived from midkine-overexpressing and/or sorafenib-treated HCC tumors showed more potent immunosuppressive activity. The potential function of midkine was also evaluated in MDSCs expanded from human PBMCs. Midkine was ectopic overexpressed in HUH-7 and SNU-449 cells (Supplementary Fig. 6A). The secreted midkine in conditional medium of HUH-7 and SNU-449 was significantly increased by midkine overexpression (Supplementary Fig. 6B). Human MDSCs are commonly marked as CD11b^+^, CD33^+^, and HLA-DR^−^. In our study, CD11b^+^CD33^+^HLA-DR^-^ MDSCs were expanded from human PBMCs via culturing with conditional medium from HUH-7 or SNU-449 cells for 5 days. In our study, conditional medium from midkine-overexpressing HUH-7 and SNU-449 cells evidently expanded more CD11b^+^CD33^+^HLA-DR^−^ MDSCs compared with conditional medium from EV-transduced cells (Supplementary Fig. 6C). The immunosuppression activity of those MDSCs were evaluated by T-cell suppression assay. T cells co-cultured with MDSCs induced by conditional medium from midkine-overexpressing HUH-7 and SNU-449 cells showed less Ki67 staining and IFN-γ production, indicating these T cells were less proliferative and cytotoxic (Supplementary Fig. 6D–F). Altogether, our data indicated that forced midkine expression stimulated immunosuppressive MDSCs accumulation in HCC tumor microenvironment.

### Midkine inhibition prevents immunosuppressive MDSCs infiltrating into HCC tumors

**Fig. 5:**
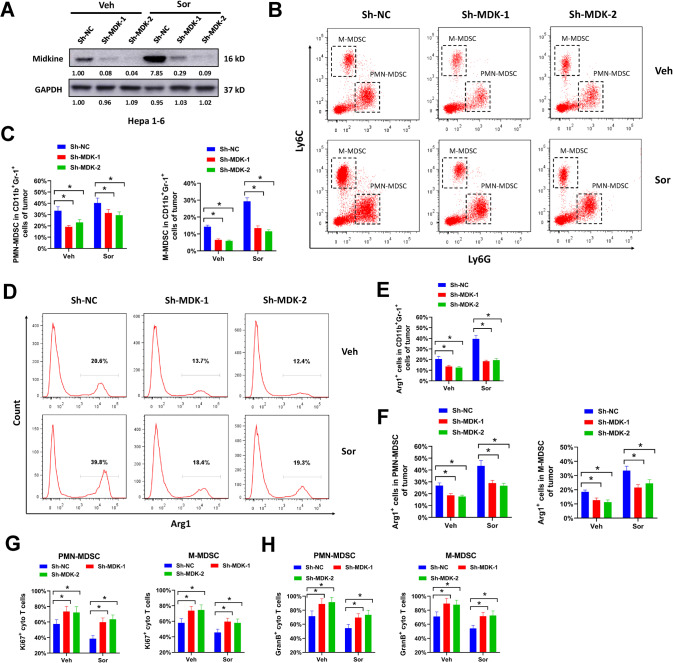
Midkine inhibition prevents immunosuppressive MDSCs infiltrating into HCC tumors. Hepa 1-6 cells transduced with Sh-MDK-1, Sh-MDK-2 or Sh-NC lentivirus were orthotopically implanted into the liver of C57BL/6 mice, then treated with sorafenib (Sor) or vehicle control (Veh) for three weeks. **A** protein expression of midkine in midkine-depleted orthotopic Hepa 1-6 tumors was validated by western blot. **B**, **C** Ly6C^int^Ly6G^+^ PMN-MDSCs and Ly6C^high^Ly6G^-^ M-MDSCs in intratumoral CD11b^+^Gr-1^+^ subset of midkine-depleted orthotopic Hepa 1-6 tumors were evaluated by flow cytometry. **D**–**F** Arg1^+^ cells in infiltrating CD11b^+^Gr-1^+^ subset (**D**–**E**) and MDSCs subset (**F**) of midkine-depleted orthotopic Hepa 1-6 tumors were evaluated by flow cytometry. **G**, **H** infiltrating PMN-MDSCs and M-MDSCs of midkine-depleted orthotopic Hepa 1-6 tumors were used for T-cell suppression assay. Ki67^+^ (**G**) and Granzyme B^+^ (**H**) cytotoxic T cells were evaluated by flow cytometry. All assays were done with at least three repeats. Data were shown as mean ± s.d., *P < 0.05.

The influence of midkine on MDSCs infiltration was further evaluated by loss-of-function assays. Short hairpin RNAs targeting mouse midkine (Sh-MDK-1 and Sh-MDK-2) were designed and introduced into Hepa 1-6 cells. Midkine expression was depleted by these two shRNAs, despite exposing to sorafenib treatment (Fig. 5A). The percentage of infiltrating MDSCs was measured. Midkine knockdown apparently decreased the percentage of infiltrating PMN-MDSCs and M-MDSCs in orthotopic Hepa 1-6 tumors with/without sorafenib treatment (Fig. 5B, C). Depletion of midkine also reduced the percentage of Arg1^+^ cells in infiltrating CD11b^+^Gr-1^+^ subset (Fig. 5D, E) and MDSCs subset (Fig. 5F). The influence of midkine inhibition on immunosuppression activity of infiltrating MDSCs was evaluated. Cytotoxic T cells co-cultured with MDSCs from midkine-depleted Hepa 1-6 tumors showed increased Ki67 and Granzyme B staining compared with cells co-cultured with MDSCs from non-targeting control (sh-NC) transduced tumors (Fig. 5G, E). This was also evaluated in MDSCs expanded from human PBMCs. Short hairpin RNAs targeting human midkine (Sh-MDK-3 and Sh-MDK-4) were designed and introduced into HUH-6 and HepG2 cells which showed high endogenous midkine expression. Midkine was successfully knocked down by these two shRNAs (Supplementary Fig. 7A). Knockdown of midkine significantly reduced the level of secreted midkine in conditional medium from HUH-6 and HepG2 cells (Supplementary Fig. 7B). Furthermore, reduced midkine secretion suppressed CD11b^+^CD33^+^HLA-DR^−^ MDSCs expansion from human PBMCs (Supplementary Fig. 7C). In T cell suppression assay, T cells co-cultured with MDSCs induced by conditional medium from midkine depleted HUH-6 and HepG2 cells exhibited more Ki67 staining and IFN-γ production, indicating these T cells were more proliferative and cytotoxic (Supplementary Fig. 7D–F). Above all, our results suggested that midkine inhibition prevented immunosuppressive MDSCs infiltrating into HCC tumors.

### Midkine inhibition enhances anti-PD-1 immunotherapy in sorafenib-treated HCC tumors

**Fig. 6:**
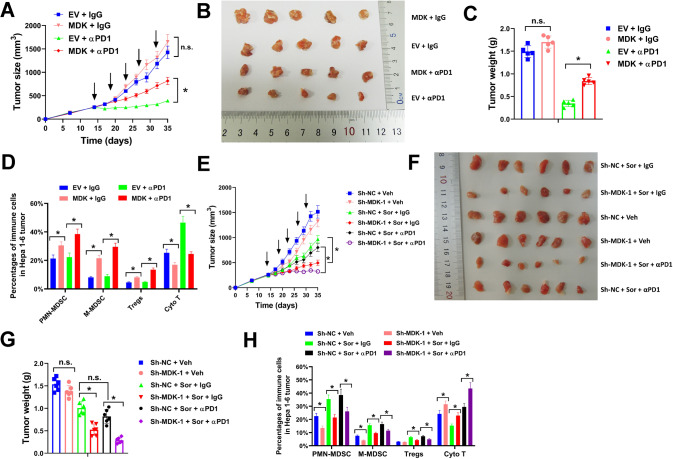
Midkine inhibition enhances anti-PD-1 immunotherapy in Sorafenib-treated HCC tumors. **A**–**D** Hepa 1-6 cells transduced with midkine expression lentivirus or EV control were subcutaneously injected into C57BL/6 mice, then treated with antibody against murine PD-1 or IgG2b isotype control as indicated. Tumor growth curves (**A**), representative images (**B**), tumor weight (**C**), and infiltrating immune cells (**D**) were shown. **E**–**H** Hepa 1-6 cells transduced with Sh-MDK-1 or Sh-NC lentivirus were subcutaneously injected into C57BL/6 mice, then treated with sorafenib, vehicle, antibody against murine PD-1, or IgG2b isotype control as indicated. Tumor growth curves (**E**), representative images (**F**), tumor weight (**G**), and infiltrating immune cells (**H**) were shown. All assays were done with at least three repeats. Data were shown as mean ± s.d., *P < 0.05.

To assess whether mikine could impinge on immunotherapy with checkpoint inhibitors, we tested anti-PD-1 antibody. Midkine overexpression showed no significant influence on growth of subcutaneous Hepa 1-6 tumors, as observed in the IgG isotype control group (Fig. 6A–C). Compared with the IgG isotype control, tumor growth, volume and weight of subcutaneous Hepa 1-6 tumors were significant reduced by anti-PD-1 antibody, however this effect was largely abolished by midkine overexpression (Fig. 6A–C). The intratumoral infiltrating immune cells were further evaluated. Forced midkine expression significantly increased the percentages of infiltrating MDSCs and Tregs, and anti-PD-1 treatment augmented this effect to some extent (Fig. 6D). In addition, anti-PD-1 treatment dramatically increased the percentage of infiltrating CD8^+^ cytotoxic T cells, however this effect was almost abrogated by midkine overexpression (Fig. 6D). Next, the influence of midkine inhibition on immunotherapy with anti-PD-1 antibody in sorafenib-treated HCC tumors was evaluated. Knockdown of midkine showed no evident influence on tumor growth of subcutaneous Hepa 1-6 tumors (Fig. 6E–G). However, midkine inhibition apparently increased the cytotoxicity of sorafenib, with reduced tumor growth, volume and weight (Fig. 6E–G). Besides, anti-PD-1 antibody showed no obvious suppression on tumor growth of sorafenib-treated tumors, but this was greatly strengthened by midkine knockdown (Fig. 6E–G). Furthermore, depletion of midkine significantly reduced the percentage of infiltrating MDSCs and Tregs, and increased the percentage of infiltrating CD8^+^ cytotoxic T cells in sorafenib-treated Hepa 1-6 tumors (Fig. 6H). Taken together, our results indicated that midkine inhibition enhanced anti-PD-1 immunotherapy in sorafenib-treated HCC tumors.

### Midkine overexpression drives multiple pathways activation and IL-10 production by MDSCs

**Fig. 7:**
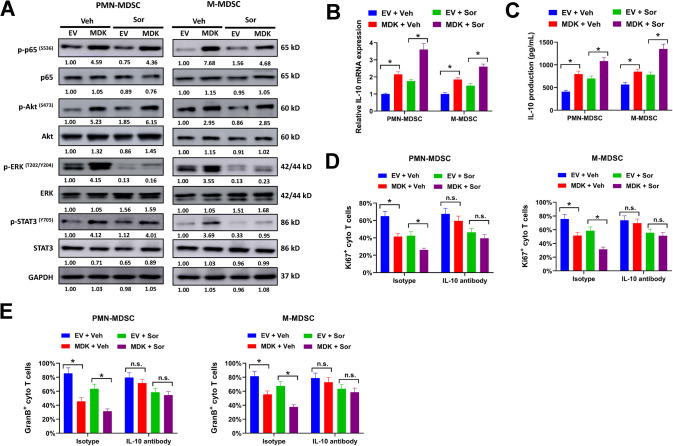
Midkine overexpression promotes multiple pathways activation and IL-10 production by MDSCs. **A**–**C** PMN-MDSCs and M-MDSCs were sorted from midkine-overexpressing or EV-transduced orthotopical Hepa 1-6 tumors with/without sorafenib treatment, then collected cell lysates for western blot (**A**). Relative IL-10 expression (**B**) and production (**C**) were evaluated by qRT-PCR and ELISA assay. **D**, **E** PMN-MDSCs, and M-MDSCs sorted from midkine-overexpressing or EV-transduced orthotopic Hepa 1-6 tumors with/without sorafenib treatment were used for T-cell suppression assay. Ki67^+^ (**D**) and Granzyme B^+^ (**E**) cytotoxic T cells were evaluated by flow cytometry. All assays were done with at least three repeats. Data were shown as mean ± s.d., *P < 0.05.

Previous studies demonstrate that midkine promotes the activation of various signaling pathways, including NF-кB, PI3K/Akt, ERK, and Notch2/Jak2/STAT3 [16, 22, 29]. In our study, MDSCs sorted from midkine-overexpressing Hepa 1-6 tumors showed increased phosphorylation of p65, Akt, ERK, and STAT3 compared with MDSCs from EV-transduced Hepa 1-6 tumors, indicating the activation of NF-кB, Akt, ERK, and STAT3 signaling by midkine-driven secretome (Fig. 7A). This was also validated in CD11b^+^CD33^+^HLA-DR^-^ MDSCs induced from human PBMCs. Conditional medium from midkine-overexpression HUH-7 and SNU-449 cells evidently promoted the phosphorylation of p65, Akt, ERK and STAT3 in CD11b^+^CD33^+^HLA-DR^−^ MDSCs compared with conditional medium form EV-transduced cells (Supplementary Fig. 8A). The immunosuppression activity of MDSCs is acted through various mechanisms. Among them, IL-10 secreted by MDSCs promotes immunosuppression via targeting a variety of immune cells [30]. In our study, IL-10 expression was significantly upregulated in sorafenib-treated HCC tumors (Fig. 1E). Moreover, MDSCs sorted from midkine-overexpressing Hepa 1-6 tumors showed elevated IL-10 expression and production compared with MDSCs derived from EV-transduced tumors (Fig. 7B, C). To clarify the potential role of IL-10 in immunosuppression activity of MDSCs, neutralizing antibodies against IL-10 were used in T-cell suppression assay. MDSCs derived from midkine-overexpressing Hepa 1-6 tumors significantly inhibited the proliferation and activation of cytotoxic T cells compared with MDSCs derived from EV-transduced tumors, but the addition of neutralizing antibodies against IL-10 largely abrogated these effects (Fig. 7D, E). Furthermore, CD11b^+^CD33^+^HLA-DR^-^ MDSCs induced by conditional medium from midkine-overexpressing HUH-7 and SNU-449 cells showed increased IL-10 expression and secretion compared with MDSCs induced by conditional medium from EV-transduced cells (Supplementary Fig. 8B and C). Moreover, midkine overexpression dramatically potentiated the inhibitory functions of CD11b^+^CD33^+^HLA-DR^-^ MDSCs on proliferation and activation of stimulated cytotoxic T cells, but this was abolished by supplement of neutralizing antibodies against IL-10 (Supplementary Fig. 8D and E). Collectively, our results proved that midkine overexpression promoted multiple pathways activation and IL-10 production by MDSCs.

## Discussion

Accumulated studies prove that midkine is involved in the malignant progression of cancers, including HCC [16, 31]. Midkine is significantly upregulated in tumor tissues and serum samples of HCC patients, and associated with poor overall survival [24, 32, 33]. Midkine is reported to protect HCC cells against TRAIL-mediated apoptosis and cadmium-induced cellular damage [34, 35]. In addition, midkine overexpression facilitates metastasis of HCC cells via increasing anoikis resistance [36]. Upregulation of midkine in HCC samples was also demonstrated by us via analyzing data from public database. What’s more, we found that sorafenib treatment promoted midkine expression and secretion by HCC cells. Sorafenib is a multikinase inhibitor with antiangiogenic property. Previous studies demonstrate that sustained sorafenib treatment reduces microvascular density and increases intratumoral hypoxia of HCC tumors [37, 38]. Corresponding with this, our data proved that sorafenib treatment decreased microvessel density, augmented intratumoral hypoxia and activated HIF-1α signaling in orthotopic HCC models. Emerging evidences indicate that midkine is vital for hypoxia-driven tumor angiogenesis [39, 40]. It is worth noting that the promoter region of midkine has a hypoxia responsive element which can binds with HIF-1α [27]. Thus, it was not surprising that midkine was significantly upregulated by sorafenib-induced hypoxia in orthotopic HCC tumors.

There are increasing evidences suggesting that persistent sorafenib treatment gives rise to an immunosuppressive tumor microenvironment. For example, sorafenib treatment increases intratumoral infiltrating of F4/80^+^ tumor-associated macrophages, CD4^+^CD25^+^FoxP3^+^ regulatory T cells, CCL2^+^/CCL17^+^ tumor-associated neutrophils and CD11b^+^Gr-1^+^ myeloid cells in human and murine HCC models [37, 41]. Besides, immunosuppressive M2 macrophages accumulate more while activated Natural Killer cells fail to proliferate and produce effector molecules in sorafenib-resistant tumors [42, 43]. Similarly, our data demonstrated that the percentages of Treg, TAM, and MDSC were increased, while cytotoxic T cells were decreased in sorafenib-treated tumors, indicating an immune-resistant HCC tumor microenvironment. To elucidate the potential molecular mechanism underlying this, we found that midkine was significantly upregulated by sorafenib treatment in HCC tumors. More importantly, forced midkine expression stimulated immunosuppressive MDSCs accumulation in HCC microenvironment, while knockdown of midkine suppressed immunosuppressive MDSCs infiltrating into HCC tumors. These results suggested midkine might partially account for sorafenib-induced immunosuppressive microenvironment. Indeed, midkine is well-characterized for its function in the immune system, including promoting immune cell chemotaxis and sculpting myeloid cell phenotype. For instance, midkine promotes neutrophils trafficking and adhesion during acute inflammation via inducing high affinity conformation of β2 integrins [19]. During ischemic renal injury, midkine deficient mice show reduced number of infiltrating neutrophils and macrophages into the tubulointerstitium and impaired induction of macrophage inflammatory protein-2 and macrophage chemotactic protein-1 [44]. Midkine is also critical for fostering the immunosuppressive microenvironment. In melanoma, midkine overexpression gives rise to an immunosuppressive tumor microenvironment via promoting intratumoral recruitment of myeloid cells and tumor-associated macrophages [22]. In ErbB pathway-mutated gallbladder cancer, upregulation of secreted midkine facilitates differentiation of immunosuppressive macrophages through biding with its receptor LRP1 [23]. Correspondingly, we found that midkine-modulated secretome promoted expansion of CD11b^+^CD33^+^HLA-DR^-^ MDSCs from human PBMCs.

In recent years, immune checkpoint inhibitors such as anti-PD-1 or anti-PD-L1 become an alternative therapeutic choice for HCC treatment, however the majority of patients do not respond to them when use as single agent [11, 45]. In contrast, the combination of anti-PD-1/anti-PD-L1 antibodies with vascular endothelial growth factor (VEGF) inhibitors has become a new first line therapy, as atezolizumab (anti-PD-L1) and bevacizumab (anti-VEGF) improves the median overall survival for more than 17 months [13]. This is consistent with the idea that checkpoint blockade may be more efficacious as combination therapy. Nevertheless, only one in four patients respond to the combination therapy, and the rest majority do not respond for unknown reasons. Sorafenib shows antiangiogenic activity by targeting a variety of tyrosine kinase receptors, including VEGF receptor 2 and 3. In our study, we found that prolonged sorafenib exposure altered HCC microenvironment towards an immune-resistant state, and anti-PD-1 antibody showed no obvious influence on tumor growth of sorafenib-treating tumors. Similarly, the response rate of anti-PD-1 drug nivolumab is lower in sorafenib experienced patient than sorafenib-naïve patients (16–19% vs. 23%) [11]. In our study, midkine was significantly induced by sorafenib in HCC tumors. Knockdown of midkine dramatically increased the inhibitory effects of anti-PD-1 antibody on sorafenib-treating tumors, and this was partially due to the destruction of sorafenib-induced immunosuppressive microenvironment. In addition, forced midkine expression apparently attenuated the suppression of anti-PD-1 antibody on HCC tumor growth. These results suggested that the immunocompromised microenvironment caused by VEGF inhibitors such as sorafenib might account for the low response rate of the combination therapy of anti-PD-1/anti-PD-L1 antibodies and VEGF inhibitors. Meanwhile, breakdown of immune-tolerant state such as midkine inhibition in our study might increase the efficiency of the combination therapy. This is also demonstrated by many other studies. For instance, inhibition of CXCR4 by AMD3100 significantly increased the efficiency of anti-PD-1 immunotherapy in sorfaenib-treated HCC tumors [37].

MDSCs are essential components of the suppressive HCC tumor microenvironment. The frequency of infiltrating MDSCs is tightly correlated with the prognosis of HCC patients and efficiency of immune checkpoint inhibitors and tyrosine kinase inhibitors. For example, Ly6G^+^ MDSCs are evidently increased in sorafenib-treated orthotopic liver tumors, and targeting Ly6G^+^ MDSCs by anti-Ly6G antibody apparently improves the inhibitory effects of sorafenib via decreasing infiltrating MDSCs and enhancing intratumoral cytotoxic T cells [46]. In HCC, CCRK promotes immunosuppressive MDSCs expansion and accumulation via NF-кB/IL-6 signaling [47]. Depletion of CCRK enhances PD-L1 blockade efficiency via reducing MDSC accumulation and increasing intratumorous cytotoxic T cells. In our study, midkine overexpression significantly increased the intratumoral accumulation of MDSCs, thus diminished the efficiency of PD-1 blockade in subcutaneous Hepa 1-6 tumors. On the contrary, midkine inhibition reduced the percentage of infiltrating MDSCs in sorafenib-treated Hepa 1-6 tumors, thus enhanced the inhibitory effects of anti-PD-1 antibody. MDSCs are generated and activated through a complicated process involving multiple signaling pathways, such as STAT3, NF-кB and ERK [48]. In our study, we found that MDSCs from midkine-overexpression HCC tumors showed increasing activation of NF-кB, Akt, ERK, and STAT3 signaling. Moreover, IL-10 production by MDSCs was evidently increased by midkine overexpression. IL-10 is one of the several MDSCs-secreted immunosuppressive effectors [49, 50]. In our study, neutralizing antibodies against IL-10 largely abrogated the inhibitory effects of MDSCs on proliferation and activation of cytotoxic T cells. These results partially explained the potential influence of midkine on generation and activation of MDSCs in HCC tumors.

In summary, we found that sorafenib treatment increased intratumoral hypoxia and altered HCC microenvironment towards an immune-resistant state in mouse models. Sorafenib treatment facilitated midkine expression and secretion by HCC cells. Moreover, midkine overexpression stimulated immunosuppressive MDSC accumulation in HCC tumor microenvironment, while midkine inhibition exhibited opposite effects. In addition, midkine inhibition enhanced anti-PD-1 immunotherapy in sorafenib-treated HCC tumors. Midkine overexpression promoted multiple pathways activation and IL-10 secretion by MDSCs. Our data provided a novel role of midkine in sorafenib-induced immunosuppressive microenvironment. Midkine might be a potential target for the combination of anti-PD-1 immunotherapy in HCC patients.

## Materials and methods

### Patient samples

The collection and use of human samples were approved by the Ethics Committee of the First Hospital of Jilin University. The study was performed according to the guidelines with the Declaration of Helsinki and Ethics Committee of the First Hospital of Jilin University. Informed consents were obtained from all enrolled participants. Four pairs of HCC specimens and adjacent normal tissues were collected from the First Hospital of Jilin University between March 2019 and June 2019. Ten blood samples from healthy donors were collected, then peripheral blood mononuclear cells (PBMCs) were isolated by Ficoll reagents (Sigma, USA) according to the manufacturers’ instructions.

### Cell culture and reagents

Murine HCC cell lines Hepa 1-6 and Hepa 1c1c7 and Human HCC cell lines HUH-7, SNU-449, HUH-6, and HepG2 were maintained in Dulbecco’s modified Eagle’s medium (DMEM, Gibco, USA) medium supplemented with 10% fetal bovine serum (FBS, Gibco, USA), 100 units/mL penicillin and streptomycin (Gibco, USA). Sorted MDSCs were cultured and expanded by RPMI medium (Gibco, USA) supplemented with 10% FBS, 100 units/mL penicillin and streptomycin (Gibco, USA), 40 ng/mL GM-CSF (Peprotech, Germany) and 40 ng/mL IL-6 (Peprotech, Germany). Cells were cultured in a humidified atmosphere at 37 ˚C with 5% CO_2_. Sorafenib (Selleck Chemicals, USA) was dissolved in dimethylsulfoxide (DMSO), thus DMSO was used as vehicle control.

### Plasmid constructs

Human or mouse midkine expression lentivirus vector was constructed by cloning the coding sequence of human or mouse midkine into the pCDH lentivirus vector (System Biosciences #CD510B). The empty pCDH lentivirus vector was used as empty vector control (EV). To deplete midkine expression, short hairpin RNAs targeting mouse midkine (Sh-MDK-1 and Sh-MDK-2) or human midkine (Sh-MDK-3 and Sh-MDK-4) were cloned into the pLKO.1 plasmid. The pLKO.1 plasmid inserted with a non-targeting sequences was used as non-targeting control (Sh-NC). The shRNA sequences were listed in Supplementary Table 2.

### Quantitative real-time polymerase chain reaction (qRT-PCR)

Total RNAs from tissue samples or cell lines were extracted by the TRIzol reagent (Takara, Japan). Complementary DNA strands were reverse-transcribed by the first-strand cDNA synthesis kit (Takara, Japan). SYBR Green PCR Master Mix (Applied Biosystems, USA) were used for qRT-PCR analysis on the ViiATM7/QuantStudio 7 Flex Real Time PCR System (Applied Biosystems, USA). GAPDH was used as internal control. Relative gene expression was calculated by the 2^−ΔΔCq^ method. The primers used for qRT-PCR analysis were listed in Supplementary Table 3.

### Western blot

Protein lysates from tissues samples or culture cells were prepared using RIPA lysis buffer (Beyotime, China) supplemented with protease inhibitors (Beyotime, China). Bradford reagent (Sigma, USA) was used to measure protein concentration. A total of 10–40 μg protein lysates were separated by 8–12% SDS-PAGE gels and transferred to polyvinylidene fluoride membranes (GE Healthcare, UK). The membranes were blocked by 5% non-fat milk, then incubated with specific first antibodies at 4 ˚C overnight and corresponding second antibodies at room temperature for 1 h. The western bands were detected by chemiluminescence imaging (Biorad, USA) using ECL kit (GE Healthcare, UK). Image J was used to quantify the protein expression. Antibodies used in our study were: HIF-1α Rabbit mAb (CST #36169, 1: 1000), GAPDH Rabbit mAb (CST #5174, 1: 5000), PD-L1 Rabbit mAb (CST #13684, 1: 1000), mouse midkine antibody (Abcam #ab281534, 1: 1000), human midkine antibody (Abcam #ab52637, 1: 1000), Erk1/2 Rabbit mAb (CST #4695, 1: 1000); Phospho-Erk1/2 (Thr202/Tyr204) Rabbit mAb (CST #4370, 1: 1000); STAT3 Mouse mAb (CST #9139, 1: 1000), Phospho-STAT3 (Tyr705) Rabbit mAb(CST #73533, 1: 1000), Akt Antibody (CST #9272, 1: 1000), Phospho-Akt (Ser473) Rabbit mAb (CST #4060, 1: 1000).

### Immunohistochemistry (IHC) staining

Tissue samples were fixed by formalin and embedded by paraffin. Tissue sections (5 μm) were deparaffinized, rehydrated, and rinsed in distilled water. Antigen retrieval was conducted using citrate buffer (pH 6.0). The endogenous peroxidase activity was quenched by 3% hydrogen peroxide. For pimonidazole staining, the sections were incubated with monoclonal mouse antibodies against pimonidazole (1:50, Hypoxyprobe Inc., USA) at 37 ˚C for 30 min. For HIF-1α staining, the sections were incubated with HIF-1α Rabbit mAb (CST #36169, 1: 100) at 4 ˚C overnight. Next, the sections were stained with horseradish peroxidase anti-rabbit antibody and detected by DAB kit (Thermo Fisher, USA). The nuclei were stained with DAPI (Sigma, USA) at room temperature for 10 min. Images were obtained by Olympus FV1000 confocal microscopy.

### Flow cytometry

The orthotopic HCC tumor tissues or subcutaneous xenografts were dissected out and minced. Then tissues were digested with 0.8 mg/mL Collagenase IV (Sigma, USA) at 37 ˚C for 1 h. The cell suspensions were filtered through 70 μm strainer and resuspended in 36% Percoll (GE Healthcare, UK). PBMCs of anonymous human healthy donors were isolated by Ficoll reagent (Sigma, USA) according to manufacturers’ instructions. For cell surface staining, 1 × 10^6^ cells were incubated with anti-Fc receptor blocking antibody (2.4G2) at 4 ˚C for 15 min. Murine samples were stained with anti-mouse CD45 APC, CD3 FITC, Gr-1 V450, CD4 PE, CD8a V450, CD25 APC-CY7, Ly6C FITC, Ly6G PECY7, CD11 PE, and F4/80 APC-CY7 from BD Bioscience (USA). Human samples were stained with anti-human CD45 APC, CD11 PE, CD33 FITC, and HLA-DR V450 from BD Bioscience (USA). For intracellular staining of Arg1, Foxp3, and Ki67, cells were fixed and permeabilized by Fixation/Permeabilization solution (BD Biosciences, USA) at 4 ˚C for 15 min. Then cells were washed and stained with anti-mouse Arg1, anti-mouse Foxp3, and anti-Ki67 from BD Bioscience (USA). Flow cytometry was performed on a B.D. Influx cell sorter (BD Bioscience, USA). Flowjo software was used to analyze the data.

### Transcriptome RNA-sequencing and TCGA data analysis

Total RNAs from sorafenib-treated or vehicle-treated orthotopic Hepa 1-6 tumors were extracted by TRIzol reagent (Thermo Fisher, USA). The sequencing library was prepared using the Illumina’s TruSeq Stranded mRNA Sample Preparation kit (Illumina, USA) and sequenced on the Illumina HiSeq 2500 platform. Fifty-base-pair sequenced reads were evaluated by the nextpresso pipeline (http://bioinfo.cnio.es/nextpresso/). The RNA sequencing data for the TCGA hepatocellular carcinoma samples were downloaded from the GDC Data Portal (https://gdac.broadinstitute.org/) and analyzed as described. FastQC v0.11.0 was used to check sequencing quality. RNA-BisSeq method was performed to filter the raw reads. The raw reads was mapped to mouse genome (GRCm38/mm10) or human genome (GRCh37/hg19) using the TopHat-2.0.10 and evaluated by HTSeq. Differentially expressed genes were analyzed by DESeq2 and defined as |Log_2_ Fold Change| ≥ 1.5 and adjusted *p* < 0.05. Samples were sequenced with three repeats.

### Tumor immune estimation resource (TIMER) and tumor-immune system interactions database (TISIDB) analysis

TIMER was used to investigate the interactions between genes and tumor immune interactions (https://cistrome.shinyapps.io/timer/). The TISIDB was used to analyze the tumor and immune system interaction (http://cis.hku.hk/TISIDB/). In the present study, the correlation of midkine expression with tumor immune infiltration was investigated by TIMER and TISIDB with the HCC database. The 22 tumor-infiltrating immune cells in HCC tissues were estimated by CIBERSORT algorithm.

### ELISA assay

The levels of midkine, IL-10, and IFN-γ in serum samples or conditional medium were evaluated by ELISA kit (Abcam # ab193761, # ab279416, # ab185986, #ab255729 and #ab174443, USA) according to manufacturer’s instructions. Briefly, samples were incubated with antibody-coated plates for 1 h at room temperature and washed for five times. Then plates were incubated with biotinylated antibody, streptavidin antibody, substrate solution, and stop solution consecutively. The absorbance at 450 nm was measured by a microplate reader. All samples were done in triplicates.

### T cell suppression assay

5 × 10^5^ CD3^+^CD8^+^ cytotoxic T cells were stimulated with CD3/CD28 dynabeads (Invitrogen, USA) in 24-well plates. At the same time, 5 × 10^5^ sorted MDSCs were added into each well in the presence of human recombinant IL-2 (R&D, USA) for 3 days. In separated experiment, cells were supplemented with IL-10 antibody (Abcam #ab133575 or #ab189392, USA) or IgG isotype control (Abcam, USA). The cells were then stained with surface markers of CD3/CD4/CD8 and intracellular protein of Ki67 (eBioscience, USA) and Granzyme B (eBioscience, USA) for flow cytometry. All samples were done with three repeats.

### Murine HCC models

Hepa 1-6 or Hepa 1c1c7 cells were stably transduced with midkine, EV, sh-MDK-1, sh-MDK-2, or sh-NC lentiviral particles as indicated for animal experiments. Orthotopic HCC models were constructed as previously described [37]. 1 × 10^6^ Hepa 1-6 or Hepa 1c1c7 cells were orthotopically implanted in the liver of 8-week-old male C57BL/6 mice. Briefly, mice were anaesthetized by ketamine/xylazine, then median liver lobe was exposed by median laparotomy. 1 × 10^6^ cells (suspended in 100 μL 1: 1 PBS and matrigel mixture) were orthotopically injected in the subcapsular region of liver by a 28 G needle. One week after implantation, these mice were randomly divided into sorafenib treatment group and vehicle group. Sorafenib treatment group received daily gavage of 50 mg/kg sorafenib (in PBS/1% Tween80), while vehicle group received equal volume of PBS/1% Tween80. Mice were sacrificed 4 weeks post tumor implantation. Orthotopic HCC tumors were dissected out, and infiltrating immune cells were isolated and evaluated by flow cytometry. In separated experiments, HCC tumor xenograft model were constructed by subcutaneously injected Hepa 1-6 cells (1 × 10^6^) into the left flank of 8-week-old male C57BL/6 mice. Two weeks after tumor injection, mice were gave daily gavage of 50 mg/kg sorafenib (in PBS/1% Tween80), antibody against murine PD-1 (100 mg intraperitoneally every 4 days for five times, BioXCell, USA) or the IgG2b control isotype (100 mg intraperitoneally every 4 days for five times, BioXCell, USA). Tumor length was measures by caliper every 3 days. At the end of drug treatment, the mice were anaesthetized by 3% isoflurane and sacrificed by broking the neck. Tumor xenografts were dissected out and weighed.

### Statistical analysis

Statistical analysis was performed by GraphPad Prism 8 (GraphPad Software, La Jolla, CA). Two-tailed Student’s *t* test and One-way ANOVA (Tukey’s post-hoc test) were used to compare difference between two or more groups respectively. Data were shown as mean ± standard deviation (*x* ± s.d). *P* < 0.05 was considered as statistically significant.

## Supplementary information

Supplementary Table 1

Supplementary Table 2

Supplementary Table 3

Supplementary Figure 1

Supplementary Figure 2

Supplementary Figure 3

Supplementary Figure 4

Supplementary Figure 5

Supplementary Figure 6

Supplementary Figure 7

Supplementary Figure 8

Supplementary figure and table legends

Original Data File

## Data Availability

The data that support the findings of this study are available on request from the corresponding author.
