# Supplementary material for: Midkine inhibition enhances anti-PD-1 immunotherapy in sorafenib-treated hepatocellular carcinoma via preventing immunosuppressive MDSCs infiltration
Source: Cell Death Discov. 2023 Mar 11;9:92. doi: 10.1038/s41420-023-01392-3 (PMC10008628; doi:10.1038/s41420-023-01392-3)
Supplement: Supplementary file 1 — Supplementary Table 1 [file 41420_2023_1392_MOESM1_ESM.docx]

**Supplementary Table 1. Dysregulated genes in orthotopic HCC tumors.**

| Gene | log2FC | P.Value |
| --- | --- | --- |
| AFP | 3.351638941 | 0.001683104 |
| PEG10 | 2.921372206 | 0.001927006 |
| SPINK1 | 2.666825199 | 0.0153985 |
| PCSK1N | 2.621466453 | 8.73E-06 |
| MDK | 2.589980065 | 0.000740269 |
| SPHK1 | 2.56984869 | 1.79E-06 |
| TRIM71 | 2.429318884 | 5.21E-09 |
| SPP1 | 2.410341458 | 0.018246222 |
| MYBL2 | 2.361021976 | 0.00012315 |
| PRAME | 2.191087347 | 6.49E-05 |
| TRNP1 | 2.160339712 | 0.000966596 |
| CD24 | 2.140443056 | 0.008662493 |
| UBD | 2.130194588 | 0.000209115 |
| PYCR1 | 2.117915248 | 0.000269723 |
| AP1M2 | 2.092546872 | 0.001077102 |
| PTTG1 | 2.055592278 | 7.94E-05 |
| RND2 | 2.028882356 | 0.000707252 |
| ZIC2 | 1.996794906 | 2.25E-05 |
| H2AW | 1.996602502 | 5.09E-05 |
| IGF2BP1 | 1.991967267 | 0.000179821 |
| CDC20 | 1.967034264 | 0.000278578 |
| DUSP9 | 1.963443601 | 0.003454032 |
| COX7B2 | 1.960546934 | 0.025652063 |
| PLEKHB1 | 1.960315937 | 7.94E-06 |
| PAGE1 | 1.920850393 | 0.007182206 |
| SELENOM | 1.880800622 | 6.58E-05 |
| CENPM | 1.874161916 | 9.47E-05 |
| BEX2 | 1.866422003 | 0.008402717 |
| UBE2C | 1.865695577 | 0.000865025 |
| TMED3 | 1.864141829 | 2.32E-06 |
| MMP9 | 1.841775812 | 0.000905331 |
| UPK3A | 1.836989618 | 0.004146804 |
| S100P | 1.818719188 | 0.029378686 |
| DKK1 | 1.783215426 | 0.018960761 |
| SPATC1L | 1.778016536 | 7.13E-05 |
| PAFAH1B3 | 1.764694126 | 4.40E-05 |
| CDT1 | 1.753704069 | 6.79E-05 |
| SLC39A4 | 1.750739276 | 0.001113508 |
| KCTD17 | 1.737057454 | 9.56E-05 |
| LTB | 1.733855417 | 0.001335976 |
| SLC22A31 | 1.729159996 | 0.004409582 |
| SLC7A10 | 1.723253981 | 6.07E-05 |
| IGF2BP2 | 1.721235812 | 0.003030814 |
| DCDC2 | 1.708635888 | 0.004179303 |
| GPC3 | 1.663212436 | 0.102860225 |
| BIRC5 | 1.659753818 | 0.001459026 |
| CCNB1 | 1.658155388 | 0.000185363 |
| TMSB10 | 1.65340221 | 0.000129084 |
| CDCA5 | 1.64567189 | 0.000142669 |
| DYDC2 | 1.613475295 | 3.51E-07 |
| ARID3A | 1.606728362 | 0.000304029 |
| LRRC1 | 1.601789352 | 0.000264491 |
| TESC | 1.591611517 | 0.011313878 |
| MSI1 | 1.585392447 | 0.004975567 |
| DEPDC1B | 1.583409293 | 0.000161575 |
| PODXL2 | 1.582267698 | 0.022116177 |
| CLEC2L | 1.58168066 | 2.68E-05 |
| SNORC | 1.570497204 | 0.002109705 |
| CCNB2 | 1.562355352 | 0.000669747 |
| AURKB | 1.559023349 | 0.000412023 |
| ANXA13 | 1.543736181 | 0.017010101 |
| SCGN | 1.542242831 | 0.029362799 |
| VEGFB | 1.534603781 | 0.005689117 |
| RECQL4 | 1.529091849 | 0.000125985 |
| CDCA7 | 1.528872111 | 0.000111327 |
| E2F1 | 1.523762888 | 0.003432083 |
| GNAZ | 1.521007344 | 0.002786547 |
| EPCAM | 1.51867431 | 0.0698007 |
| KIFC1 | 1.518501482 | 0.001815519 |
| KIF2C | 1.515998154 | 0.00060893 |
| NQO1 | 1.510874734 | 0.05681938 |
| BAAT | -1.502995106 | 0.00103264 |
| MROH2A | -1.504426864 | 0.01754654 |
| FGG | -1.504790259 | 0.006133155 |
| HBB | -1.511833494 | 0.004214396 |
| CYP4A11 | -1.521191397 | 0.003030888 |
| ANGPTL4 | -1.527101617 | 0.002337511 |
| SERPINC1 | -1.528320896 | 0.010876971 |
| IRF6 | -1.528802381 | 0.001142232 |
| APOA5 | -1.538638947 | 0.010736193 |
| DPYS | -1.544949452 | 0.007093771 |
| CYP4F11 | -1.545464771 | 0.000461624 |
| AOX1 | -1.549279178 | 0.037781213 |
| ACSM2A | -1.54952846 | 0.000944808 |
| SLC51A | -1.555146977 | 0.003688169 |
| ALDH6A1 | -1.560385877 | 0.000106444 |
| ACADL | -1.56802396 | 0.001081465 |
| SLC22A7 | -1.568373277 | 0.025235691 |
| SULT1B1 | -1.570714701 | 0.003140867 |
| F13B | -1.583945052 | 0.001504052 |
| ADH1A | -1.584542688 | 0.013186409 |
| CYP17A1 | -1.584693322 | 0.039087724 |
| SHMT1 | -1.589149565 | 6.74E-05 |
| C8A | -1.590207492 | 0.00196841 |
| DCN | -1.596684889 | 0.011970684 |
| ASPDH | -1.604174062 | 0.004375391 |
| ZNF385B | -1.613105868 | 0.000430028 |
| SLC1A1 | -1.614443259 | 0.007800206 |
| MAMDC4 | -1.614542465 | 0.003674516 |
| CES2 | -1.618277498 | 0.000939504 |
| CFHR1 | -1.618528455 | 0.013848088 |
| C8B | -1.627631512 | 0.000482858 |
| AZGP1 | -1.630713461 | 0.000447322 |
| PGLYRP2 | -1.634152467 | 0.011256409 |
| UGT2B10 | -1.634223797 | 0.018814212 |
| ACSM2B | -1.639434585 | 0.000201461 |
| CHRNA4 | -1.645097797 | 0.002377952 |
| GFRA1 | -1.651366956 | 0.002975769 |
| SDS | -1.653768993 | 0.1025305 |
| LUM | -1.660422299 | 0.023486885 |
| NAT2 | -1.668457906 | 0.009641947 |
| DAO | -1.668697935 | 0.00181642 |
| CLRN3 | -1.676662539 | 0.028372321 |
| HAO1 | -1.681776628 | 0.001542742 |
| PDK4 | -1.682118614 | 0.004420406 |
| INHBC | -1.687158399 | 0.005766783 |
| FETUB | -1.691025585 | 0.017080635 |
| CLDN2 | -1.691861819 | 0.056523661 |
| UBXN10 | -1.698913736 | 0.0010092 |
| TM6SF2 | -1.709813262 | 4.35E-05 |
| RASD1 | -1.716164132 | 0.0019962 |
| RAMP1 | -1.718758864 | 0.008506515 |
| DMGDH | -1.719191704 | 0.001707333 |
| BBOX1 | -1.720691158 | 0.009938168 |
| FABP4 | -1.724421056 | 0.001132759 |
| XDH | -1.738414648 | 0.000948147 |
| NEU4 | -1.755464341 | 0.010714651 |
| SLC46A3 | -1.762612768 | 0.004919374 |
| IGFALS | -1.778813545 | 0.017593074 |
| ANO1 | -1.779025848 | 0.001769204 |
| COL5A3 | -1.781421383 | 0.000759896 |
| BHMT | -1.785323174 | 0.012371145 |
| HPD | -1.79051808 | 0.055850199 |
| GPD1 | -1.810259684 | 0.000974403 |
| EEF1A2 | -1.829726143 | 0.085302808 |
| PLG | -1.832085774 | 0.000861218 |
| AKR7A3 | -1.83686125 | 0.004311757 |
| GLYATL1 | -1.840573699 | 0.00077141 |
| AR | -1.854394234 | 9.04E-05 |
| ACSM5 | -1.859417746 | 0.00115301 |
| GSTA1 | -1.861473244 | 0.006141048 |
| SLC27A5 | -1.869775428 | 0.001406131 |
| SULT2A1 | -1.875044172 | 0.003164753 |
| ABCB4 | -1.88560255 | 0.000263453 |
| CTH | -1.896420176 | 0.001753724 |
| CYP2A7 | -1.896452668 | 0.065340933 |
| CFHR3 | -1.904604667 | 0.006946501 |
| C4BPA | -1.90955858 | 0.005325526 |
| G0S2 | -1.911520041 | 0.003972718 |
| ETNPPL | -1.932081267 | 0.004933426 |
| UGT1A4 | -1.935511355 | 0.034795514 |
| CYP2C9 | -1.939389937 | 0.006480814 |
| TTC36 | -1.94667244 | 0.017660774 |
| C7 | -1.956158162 | 0.011727293 |
| CPS1 | -1.958612158 | 0.021245158 |
| FOS | -1.958709987 | 0.001054624 |
| PON1 | -1.961852475 | 0.001265967 |
| PDZK1IP1 | -1.961884675 | 0.024326572 |
| NR1I2 | -1.978463381 | 0.000157429 |
| FMO3 | -1.986880277 | 0.001514564 |
| GLYAT | -2.006005983 | 0.012260602 |
| PCK1 | -2.007594332 | 0.010678963 |
| FGF21 | -2.046400449 | 0.002973434 |
| SEC14L2 | -2.055049178 | 0.000182706 |
| ALDOB | -2.074731326 | 0.002857327 |
| GHR | -2.087789691 | 5.10E-06 |
| MOGAT2 | -2.104062628 | 0.001513539 |
| HSD17B6 | -2.111771942 | 0.002331273 |
| UGT2B17 | -2.182848976 | 0.002925963 |
| GREM2 | -2.184025681 | 0.001013791 |
| SRD5A2 | -2.221542489 | 0.000652472 |
| FNDC5 | -2.235697732 | 0.002735349 |
| GYS2 | -2.249847903 | 0.001417832 |
| TAT | -2.271375771 | 0.0098958 |
| AFM | -2.273368465 | 0.000467084 |
| CYP4F2 | -2.287794645 | 0.000152077 |
| RTP3 | -2.30855428 | 0.000628172 |
| AQP9 | -2.311633783 | 0.003763491 |
| ADH4 | -2.328169508 | 0.023056295 |
| G6PC1 | -2.336867866 | 0.000156597 |
| MFSD2A | -2.344369001 | 0.001596334 |
| CFHR5 | -2.363420028 | 0.001699037 |
| SLC28A1 | -2.372232436 | 0.000144539 |
| GSTA2 | -2.406728115 | 0.005687444 |
| HSD17B13 | -2.416882624 | 0.033111556 |
| HSD11B1 | -2.472560319 | 0.012013943 |
| C6 | -2.476082304 | 2.87E-05 |
| CYP2C8 | -2.499449503 | 0.000927593 |
| ADH1C | -2.547534578 | 0.003270069 |
| GSTM1 | -2.561275695 | 0.024969212 |
| ANXA10 | -2.5834561 | 1.25E-05 |
| SLC22A1 | -2.586098286 | 0.007853755 |
| THRSP | -2.625733622 | 0.011501149 |
| CYP1A2 | -2.682158572 | 0.012072582 |
| HAO2 | -2.690212775 | 0.001079516 |
| CYP3A4 | -2.769466067 | 0.034733846 |
| UGT2B15 | -2.793236312 | 0.000154386 |
| ADH1B | -2.829636338 | 4.04E-05 |
| SLC10A1 | -2.938739878 | 0.000308539 |
| CYP8B1 | -3.058802785 | 0.00035389 |
| CFHR4 | -3.133924692 | 3.65E-06 |
| CYP2A6 | -4.183173779 | 0.000131344 |
