# Supplementary material for: Midkine inhibition enhances anti-PD-1 immunotherapy in sorafenib-treated hepatocellular carcinoma via preventing immunosuppressive MDSCs infiltration
Source: Cell Death Discov. 2023 Mar 11;9:92. doi: 10.1038/s41420-023-01392-3 (PMC10008628; doi:10.1038/s41420-023-01392-3)
Supplement: Supplementary file 2 — Supplementary Table 2 [file 41420_2023_1392_MOESM2_ESM.docx]

| **Name** | **Sequence** |
| --- | --- |
| Sh-MDK-1 | CCCAAGATATAACCCACCAGT |
| Sh-MDK-2 | GCCGACTGCAAATACAAGTTT |
| Sh-MDK-3 | CGACTGCAAGTACAAGTTTGA |
| Sh-MDK-4 | CAAGACCAAAGCAAAGGCCAA |
| Sh-NC | ACGGAGGCTAAGCGTCGCAA |

**Supplementary Table 2. shRNA sequences targeting midkine.**
