# Supplementary material for: Midkine inhibition enhances anti-PD-1 immunotherapy in sorafenib-treated hepatocellular carcinoma via preventing immunosuppressive MDSCs infiltration
Source: Cell Death Discov. 2023 Mar 11;9:92. doi: 10.1038/s41420-023-01392-3 (PMC10008628; doi:10.1038/s41420-023-01392-3)
Supplement: Supplementary file 3 — Supplementary Table 3 [file 41420_2023_1392_MOESM3_ESM.docx]

Supplementary Table 3. Primers of indicated genes used in qRT-PCR.

| Gene name | Sense primer | Anti-senser primer |
| --- | --- | --- |
| Human midkine | CCTGCAACTGGAAGAAGGAG | CTGGCACTGAGCATTGTAGC |
| Human GLUT-1 | GCAGGAGATGAAGGAAGAG | TCGTGGAGTAATAGAAGACAG |
| Human CA-9 | GAGAAGGCAGCACAGAAG | GCAGGACAGGACAGTTAC |
| Human CXCR4 | CAGTGAGGCAGATGACAG | ACAATACCAGGCAGGATAAG |
| Human MDR-1 | GATGATGACACGGAGAAGT | GGTGGTTAATGCTGAGGTA |
| Human VEGF | GGCAGAATCATCACGAAGT | CACAGGATGGCTTGAAGAT |
| Human Il10 | AGCCTACATGACAATGAAGA | GGTTGAGGTATCAGAGGTAAT |
| Murine midkine | GCACCTCCAAGACCAAGT | ACAGGCGTGATTGACAGA |
| Murine Pdl1 | GCATTATATTCACAGCCTGC | CCCTTCAAAAGCTGGTCCTT |
| Murine Tgfb | GACCGCAACAACGCCATCTA | GGCGTATCAGTGGGGGTCAG |
| Murine Il10 | TGGCCCAGAAATCAAGGAGC | CAGCAGACTCAATACACACT |
| Murine Il13 | GAGCAACACTCCTGTCTG | CATTCACTACACATCACCTTG |
| Murine Arg1 | AACACGGCAGTGGCTTTAACC | GGTTTTCATGTGGCGCATTC |
| Murine Nos2 | CGAAACGCTTCACTTCCAA | TGAGCCTATATTGCTGTGGCT |
