# Supplementary figures and images for: Midkine inhibition enhances anti-PD-1 immunotherapy in sorafenib-treated hepatocellular carcinoma via preventing immunosuppressive MDSCs infiltration
Source: Cell Death Discov. 2023 Mar 11;9:92. doi: 10.1038/s41420-023-01392-3 (PMC10008628; doi:10.1038/s41420-023-01392-3)

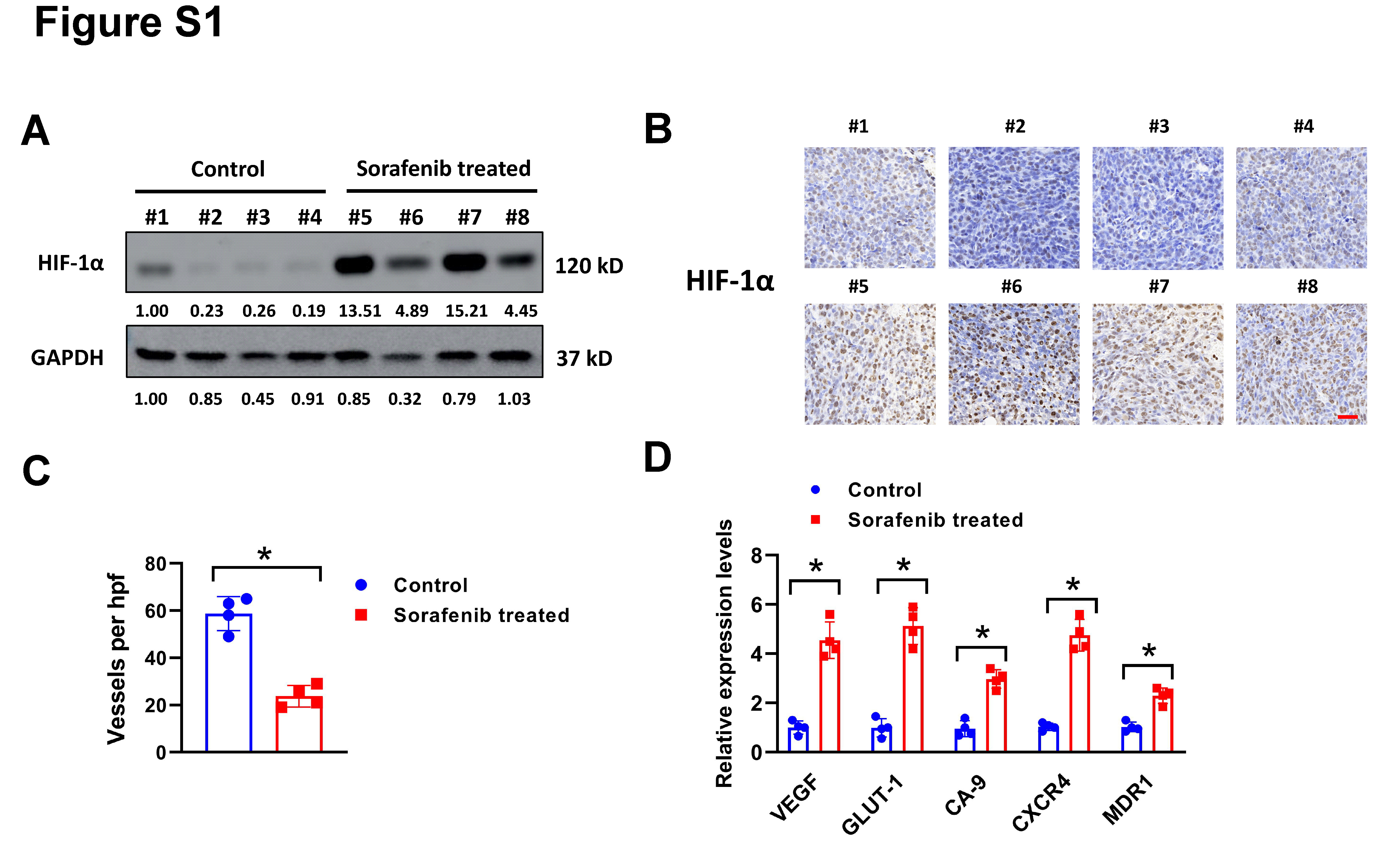

Supplement: Supplementary file 4 — Supplementary Figure 1 [file 41420_2023_1392_MOESM4_ESM.jpg]

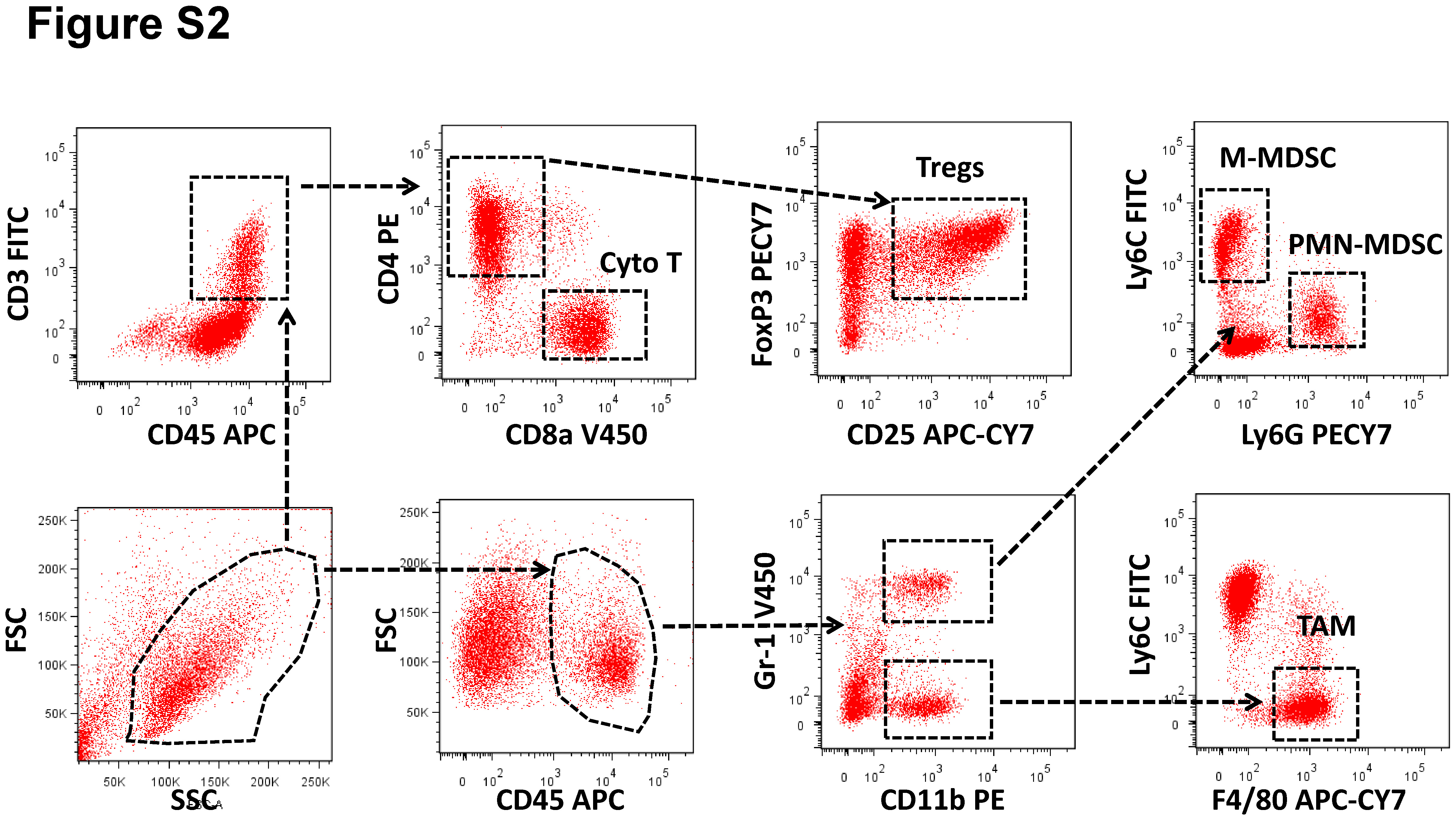

Supplement: Supplementary file 5 — Supplementary Figure 2 [file 41420_2023_1392_MOESM5_ESM.jpg]

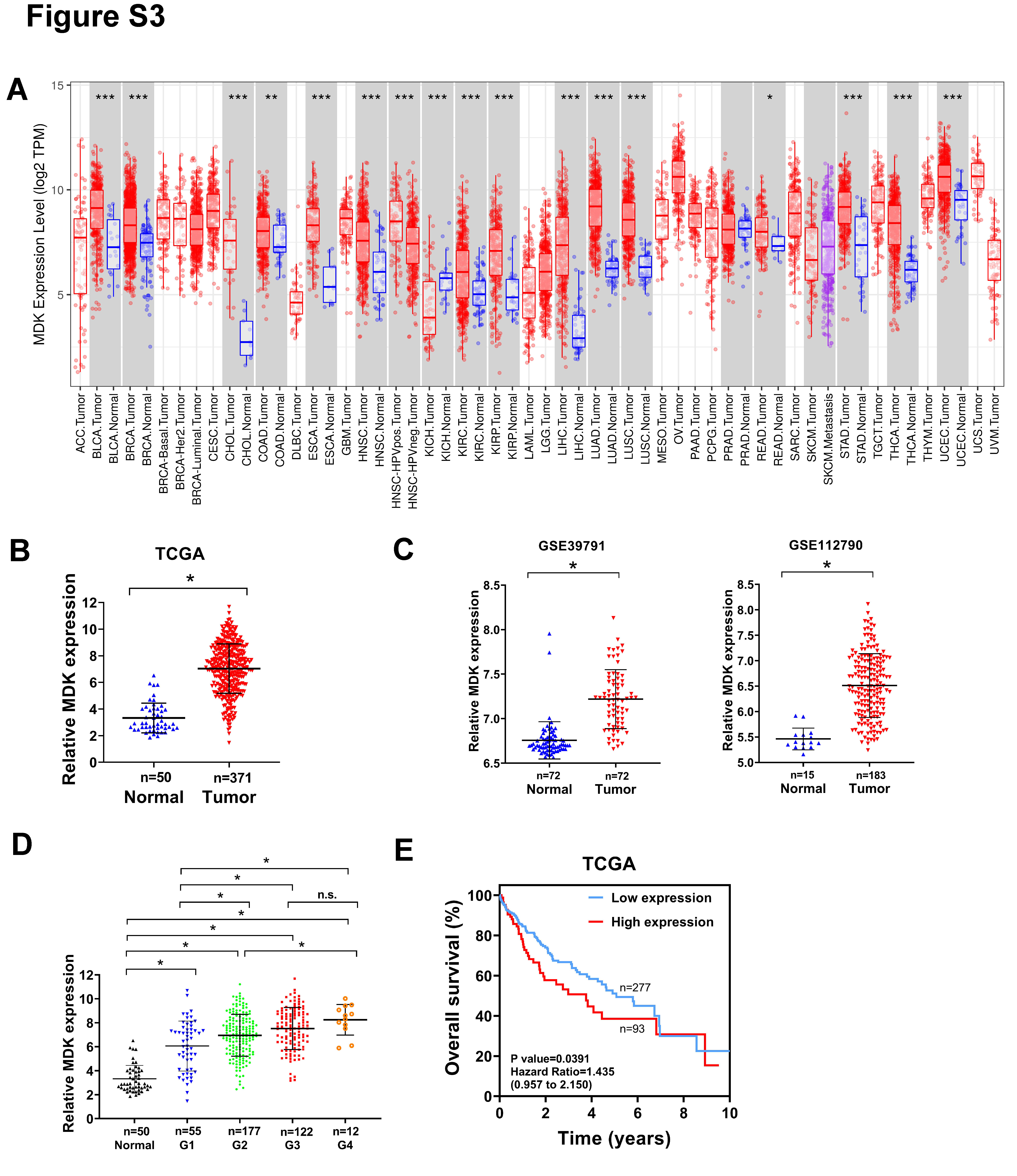

Supplement: Supplementary file 6 — Supplementary Figure 3 [file 41420_2023_1392_MOESM6_ESM.jpg]

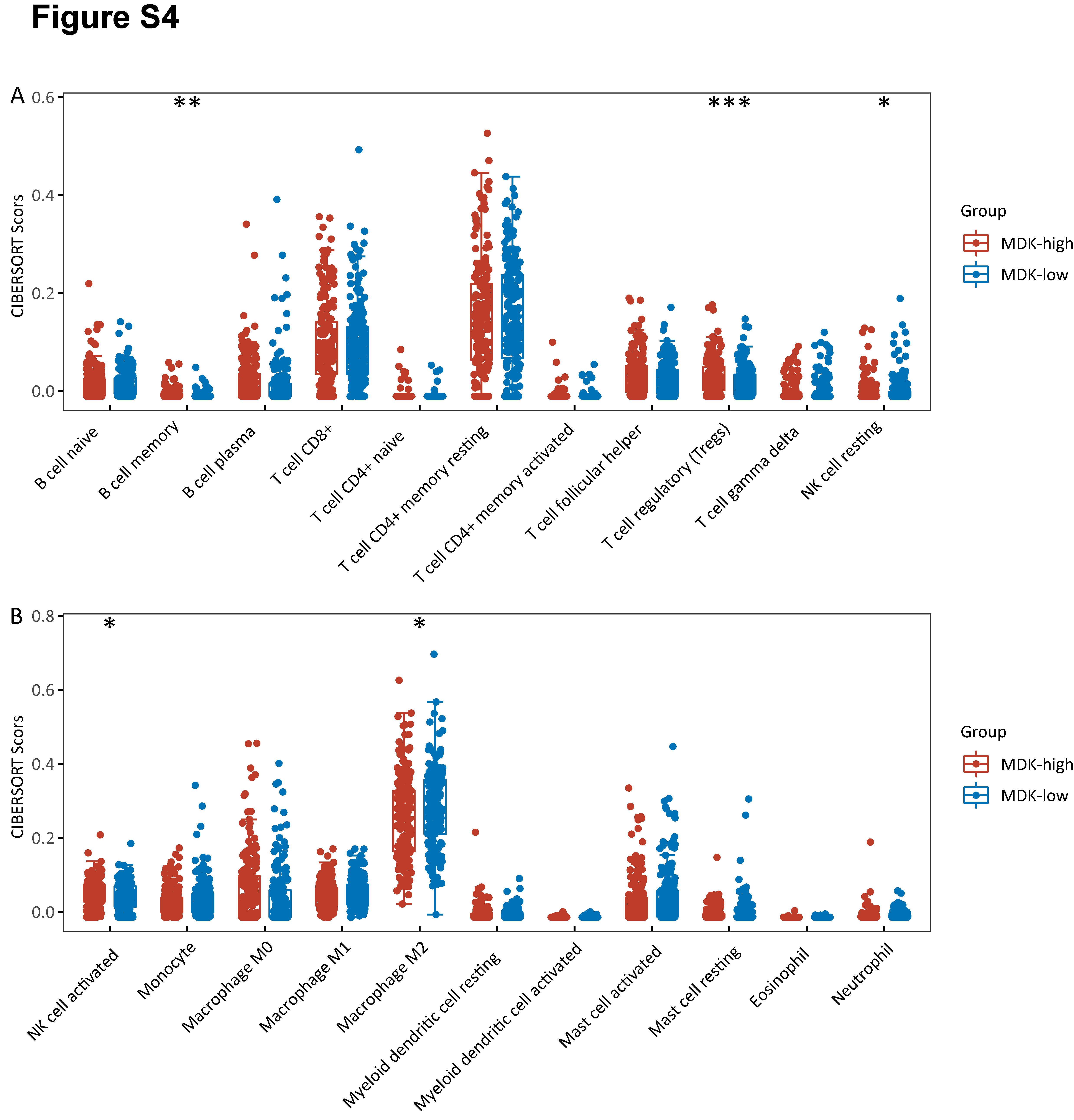

Supplement: Supplementary file 7 — Supplementary Figure 4 [file 41420_2023_1392_MOESM7_ESM.jpg]

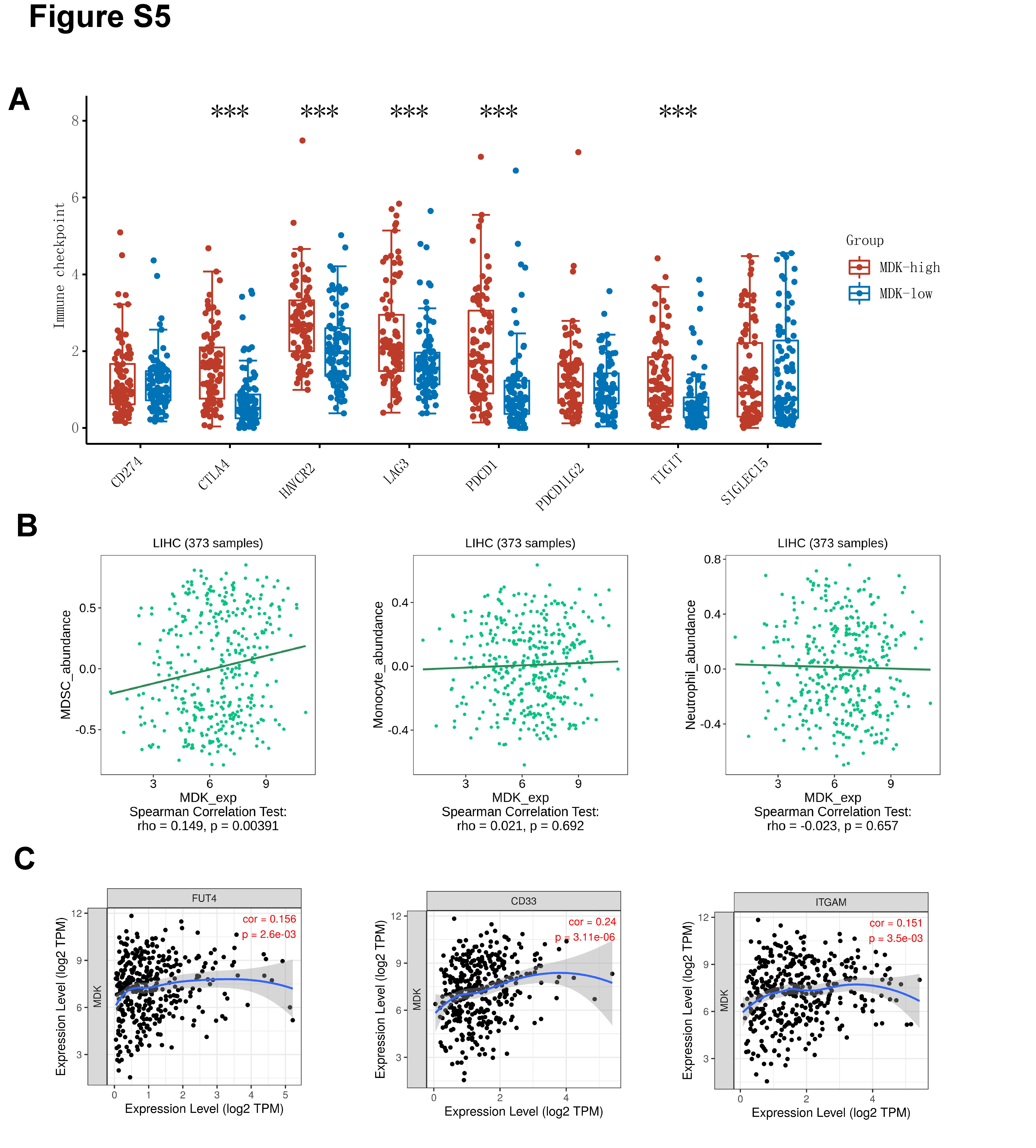

Supplement: Supplementary file 8 — Supplementary Figure 5 [file 41420_2023_1392_MOESM8_ESM.jpg]

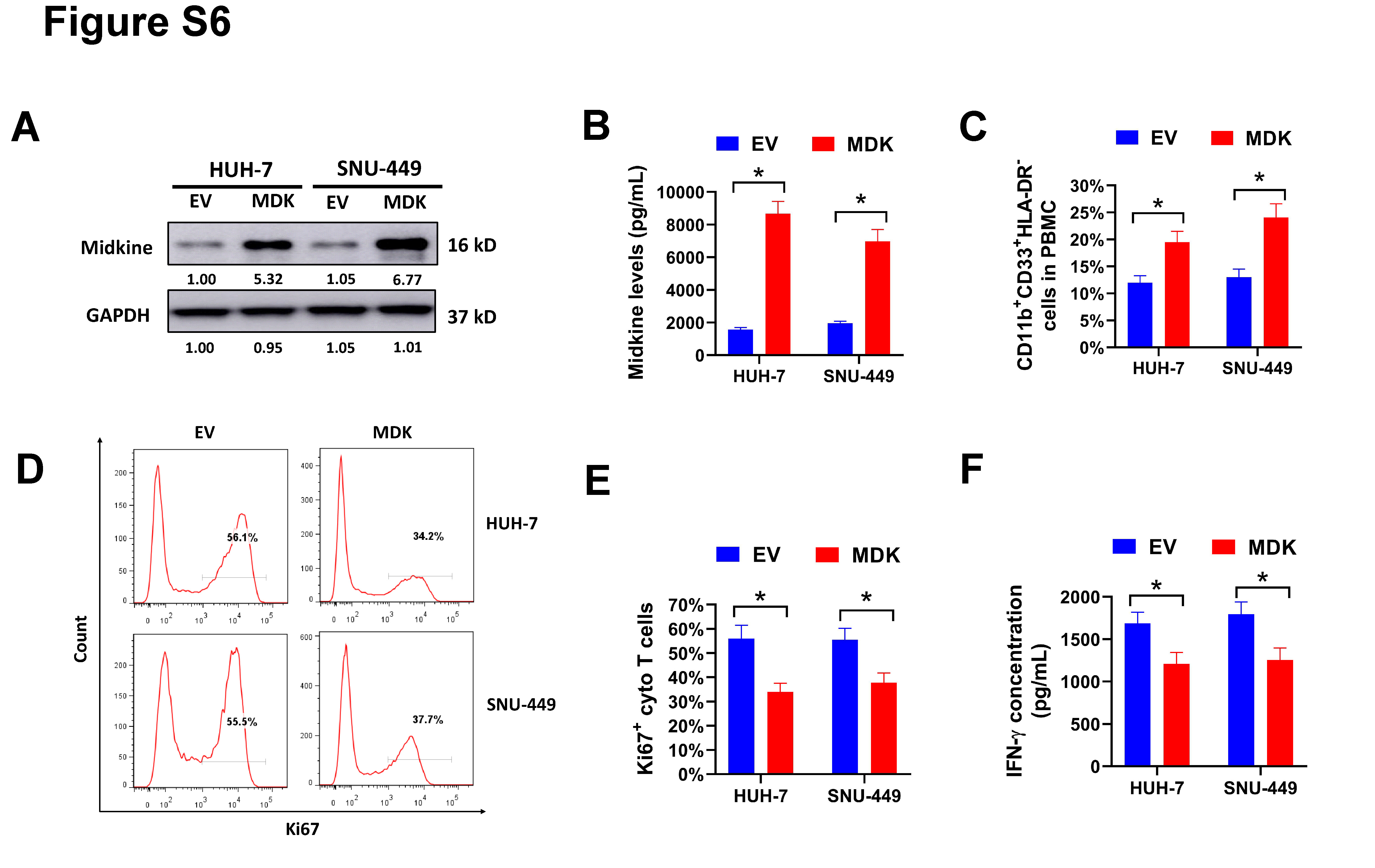

Supplement: Supplementary file 9 — Supplementary Figure 6 [file 41420_2023_1392_MOESM9_ESM.jpg]

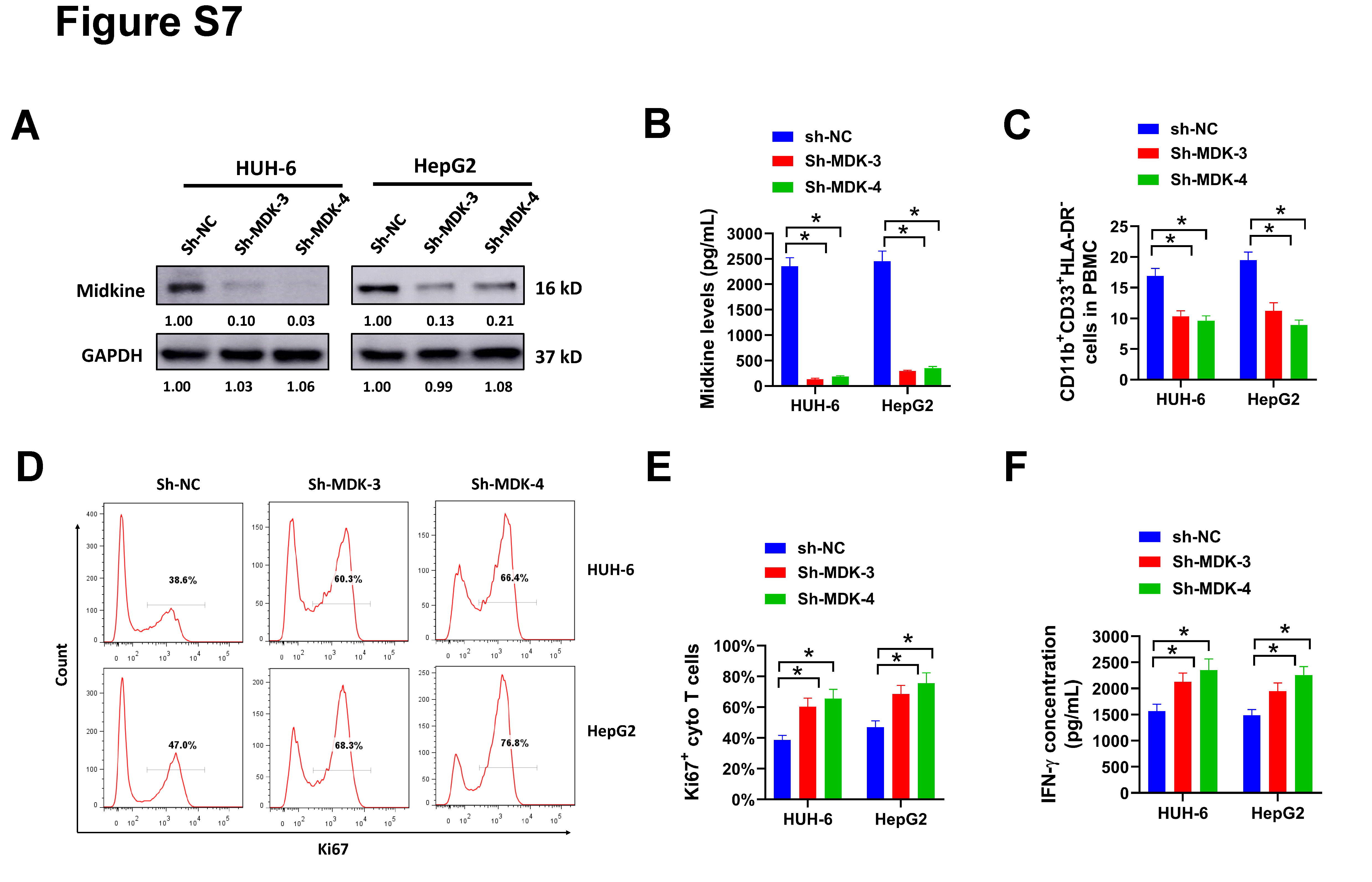

Supplement: Supplementary file 10 — Supplementary Figure 7 [file 41420_2023_1392_MOESM10_ESM.jpg]

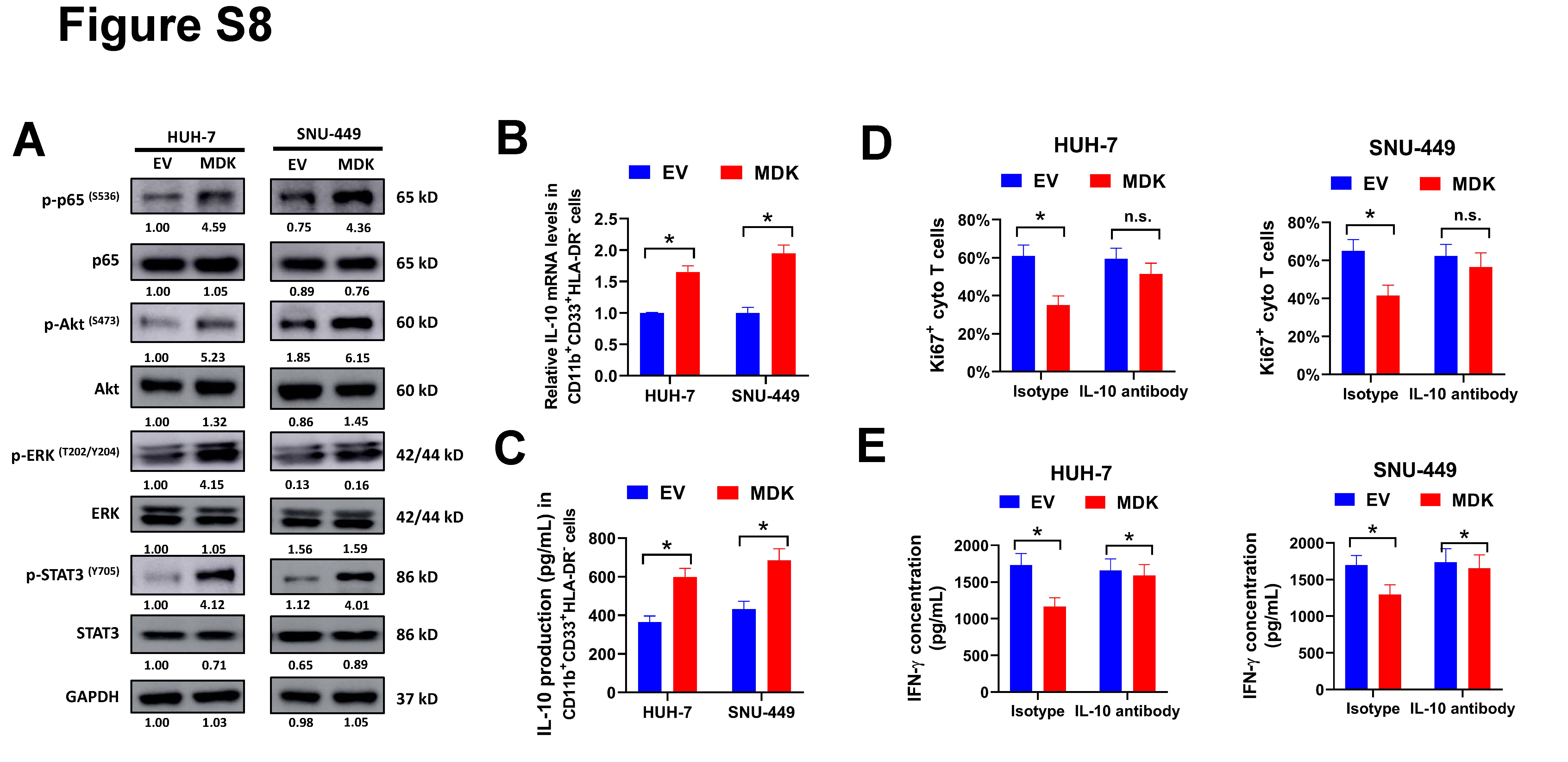

Supplement: Supplementary file 11 — Supplementary Figure 8 [file 41420_2023_1392_MOESM11_ESM.jpg]
