# Supplementary material for: Midkine inhibition enhances anti-PD-1 immunotherapy in sorafenib-treated hepatocellular carcinoma via preventing immunosuppressive MDSCs infiltration
Source: Cell Death Discov. 2023 Mar 11;9:92. doi: 10.1038/s41420-023-01392-3 (PMC10008628; doi:10.1038/s41420-023-01392-3)
Supplement: Supplementary file 12 — Supplementary figure and table legends [file 41420_2023_1392_MOESM12_ESM.docx]

Figure S1. Sorafenib treatment increases intratumoral hypoxia in HCC patients. A-B, HIF-1α expression in sorafenib-treated HCC patients (#5, #6, #7 and #8) or non-treated HCC patients (#1, #2, #3 and #4) was evaluated by western blot (A) and IHC staining (B). C, vessel density of sorafenib-treated HCC patients or non-treated HCC patients was shown. D, relative expression of indicated genes in sorafenib-treated HCC patients or non-treated HCC patients was evaluated by qRT-PCR. All assays were done with at least three repeats. Data were shown as mean ± s.d., **P* < 0.05.

Figure S2. Gating strategy and representative plots of infiltrating immune cell populations in HCC tumors.

Figure S3. Midkine is upregulated in HCC patients. A, midkine expression in pan-cancer was evaluated by TIMER database. B-C, relative midkine expression in HCC patients was evaluated in TCGA and GEO database (GSE39791 and GSE112790). D, relative midkine expression in TCGA-HCC subset according to tumor stages. E, Kaplan-Meier survival analysis of TCGA-HCC subset according to midkine expression. All assays were done with at least three repeats. Data were shown as mean ± s.d., **P* < 0.05.

Figure S4. Correlation of Midkine expression with infiltration immune cell populations in HCC patients from TCGA. A-B，the 22 tumor-infiltrating immune cells in HCC samples were estimated using the CIBERSORT algorithm according to midkine expression.

Figure S5. Correlation of Midkine expression with markers of immunosuppressive cells and molecules. A，correlation of midkine expression with immune checkpoint molecules in HCC patients. B, association of midkine expression with tumor-infiltrating immune cells in HCC samples was evaluated by TISIDB database. C, correlation of midkine expression with immune makers (CD33, ITGAM and FUT4) in HCC patients. ****P* < 0.001.

Figure S6. Hepatocellular Midkine secretion induces T cell-suppressive MDSCs expansion from human PBMCs. A, HUH-7 and SNU-449 cells were transduced with midkine expression lentivirus or EV control, then collected lysates for western blot. B, secreted midkine in conditional medium of HUH-7 and SNU-449 cells was evaluated by ELISA assay. C, human PBMCs were cultured with conditional medium from mdikine-overexpressing or EV-transduced HUH-7 and SNU-449 cells for 5 days, then CD11b^+^CD33^+^HLA-DR^-^ MDSCs were evaluated by flow cytometry. D-F, CD11b^+^CD33^+^HLA-DR^-^ MDSCs expanded by conditional medium from HUH-7 and SNU-449 were used for T-cell suppression assay. Ki67^+^ cells (D-E) and IFN-γ production (F) in cytotoxic T cells were evaluated by flow cytometry and ELISA assay. All assays were done with at least three repeats. Data were shown as mean ± s.d., **P* < 0.05.

Figure S7. Midkine knockdown suppresses T cell-suppressive MDSCs expansion from human PBMCs. A, HUH-6 and HepG2 cells were transduced with Sh-MDK-3, Sh-MDK-4 or Sh-NC, then collected lysates for western blot. B, secreted midkine in conditional medium of HUH-6 and HepG2 was evaluated by ELISA assay. C, human PBMCs were cultured with conditional medium from Sh-MDK-3, Sh-MDK-4 or Sh-NC transduced HUH-6 and HepG2 cells for 5 days, then CD11b^+^CD33^+^HLA-DR^-^ MDSCs were evaluated by flow cytometry. D-F, CD11b^+^CD33^+^HLA-DR^-^ MDSCs expanded by conditional medium of HUH-6 and HepG2 cells were used for T-cell suppression assay. Ki67^+^ cells (D-E) and IFN-γ production (F) in cytotoxic T cells were evaluated by flow cytometry and ELISA assay. All assays were done with at least three repeats. Data were shown as mean ± s.d., **P* < 0.05.

Figure S8. Forced Midkine expression induces IL-10 production in T cell-suppressive MDSCs from human PMBCs. A-C, CD11b^+^CD33^+^HLA-DR^-^ MDSCs were expanded by conditional medium from mdikine-overexpressing or EV-transduced HUH-7 and SNU-449 cells for 5 days, then collected cell lysates for western blot analysis (A). Relative IL-10 expression (B) and production (C) were evaluated by qRT-PCR and ELISA assay. D-E, CD11b^+^CD33^+^HLA-DR^-^ MDSCs expanded from mdikine-overexpressing or EV-transduced HUH-7 and SNU-449 cells were used for T-cell suppression assay. Ki67^+^ cells (D) and IFN-γ production (E) in cytotoxic T cells were evaluated by flow cytometry and ELISA assay. All assays were done with at least three repeats. Data were shown as mean ± s.d., **P* < 0.05.

Supplementary Table 1. Dysregulated genes in orthotopic HCC tumors.

Supplementary Table 2. shRNA sequences targeting midkine.

Supplementary Table 3. Primers of indicated genes used in qRT-PCR.
