# Supplementary material for: Midkine inhibition enhances anti-PD-1 immunotherapy in sorafenib-treated hepatocellular carcinoma via preventing immunosuppressive MDSCs infiltration
Source: Cell Death Discov. 2023 Mar 11;9:92. doi: 10.1038/s41420-023-01392-3 (PMC10008628; doi:10.1038/s41420-023-01392-3)
Supplement: Supplementary file 13 — Original Data File [file 41420_2023_1392_MOESM13_ESM.pdf]

Fig 1A

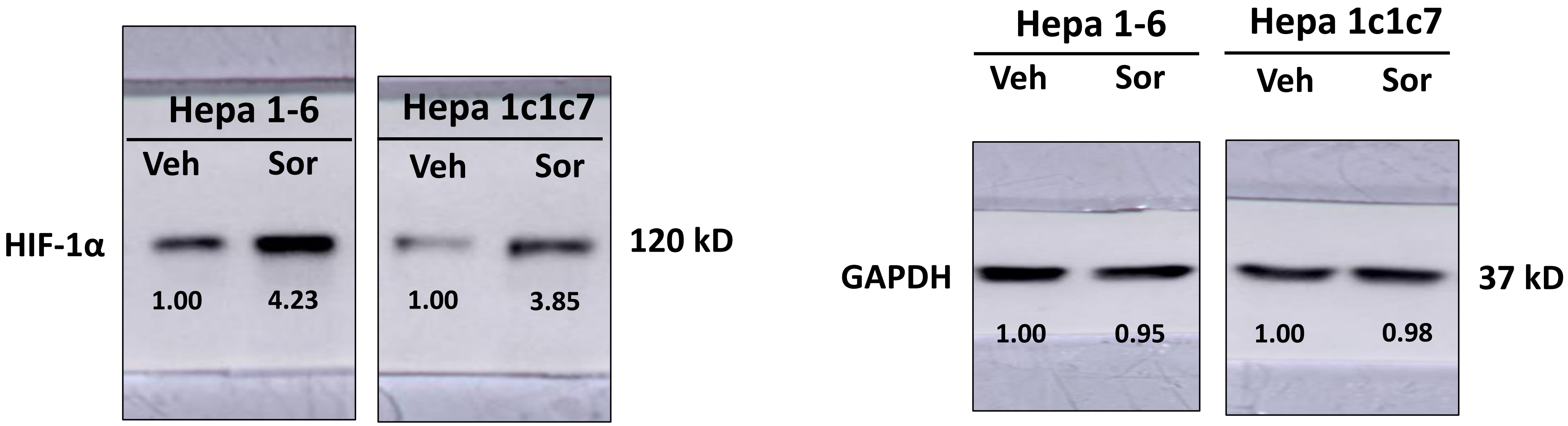

Hepa 1-6
Veh
Sor
Hepa 1c1c7
Veh
Sor
HIF-1α
120 kD
1.00
4.23
1.00
3.85
Hepa 1-6
Hepa 1c1c7
Veh
Sor
Veh
Sor
GAPDH
37 kD
1.00
0.95
1.00
0.98

Fig 1F

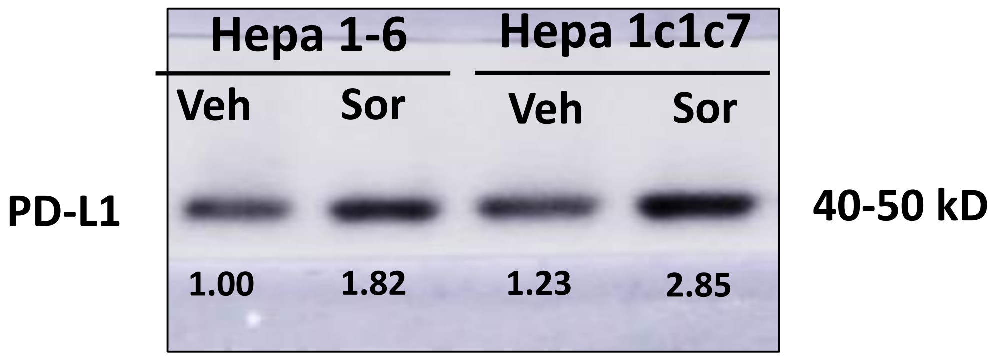
Hepa 1-6
Hepa 1c1c7
Veh
Sor
Veh
Sor
PD-L1
40-50 kD
1.00
1.82
1.23
2.85

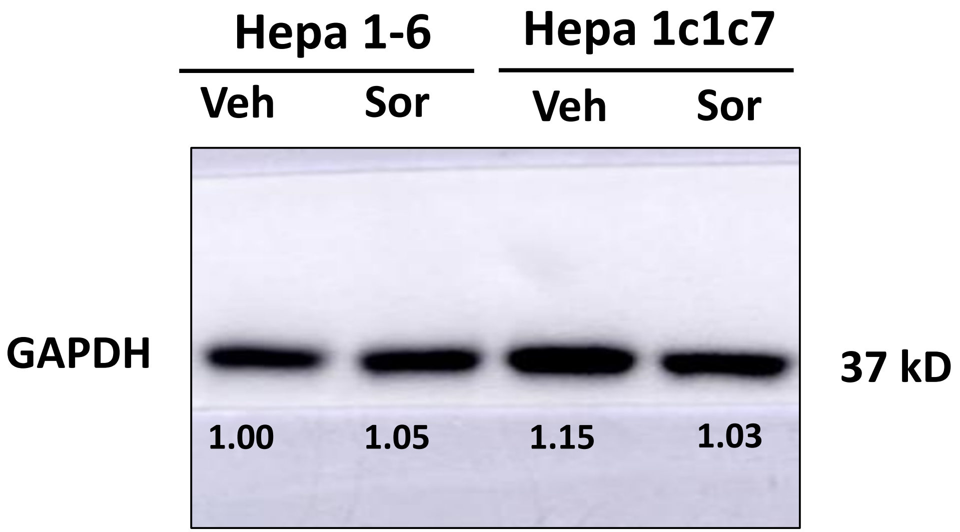
Hepa 1-6
Hepa 1c1c7
Veh
Sor
Veh
Sor
GAPDH
37 kD
1.00
1.05
1.15
1.03

Fig 2C

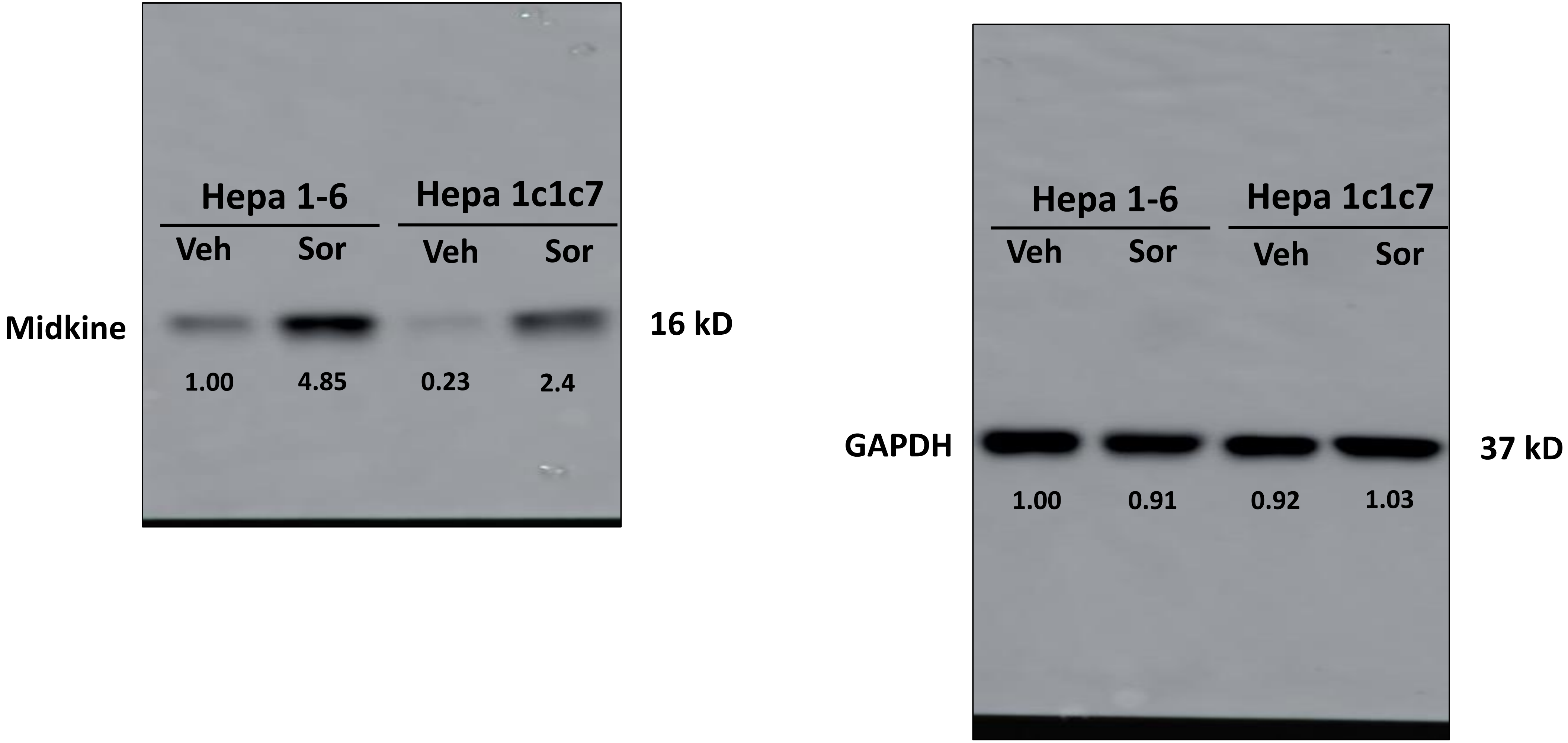

Hepa 1-6
Hepa 1c1c7
Veh
Sor
Veh
Sor
Midkine
16 kD
1.00
4.85
0.23
2.4
Hepa 1-6
Hepa 1c1c7
Veh
Sor
Veh
Sor
GAPDH
37 kD
1.00
0.91
0.92
1.03

Fig 2E

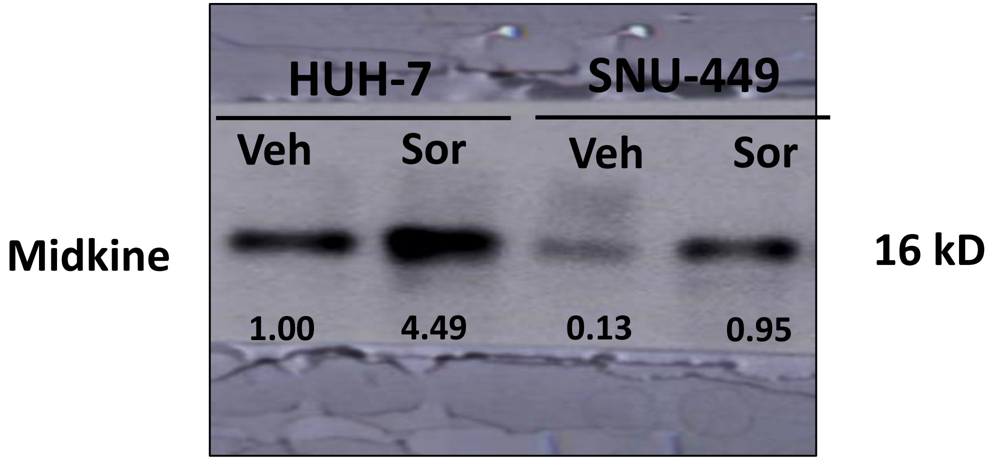
HUH-7
SNU-449
Veh
Sor
Veh
Sor
Midkine
16 kD
1.00
4.49
0.13
0.95

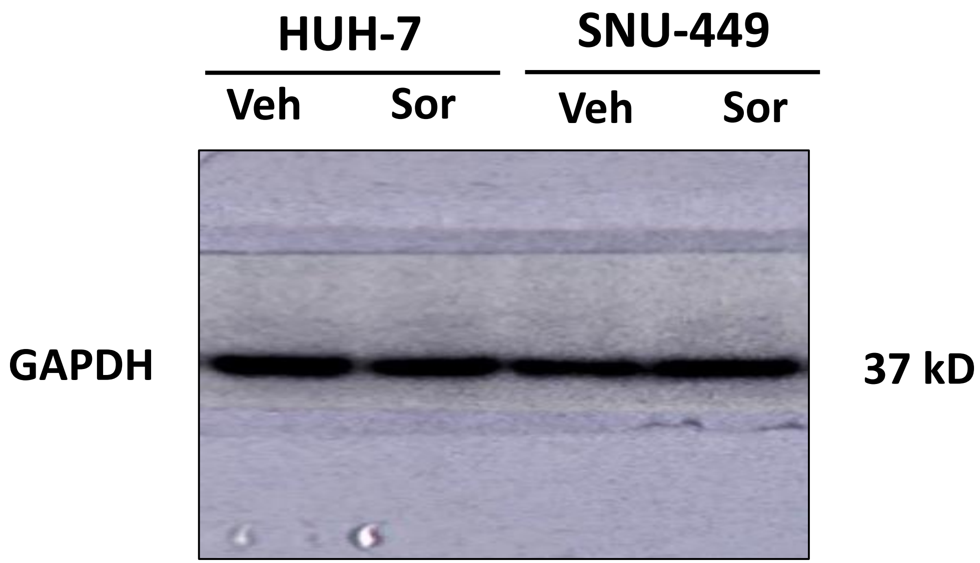
HUH-7
SNU-449
Veh
Sor
Veh
Sor
GAPDH
37 kD

Fig 3A

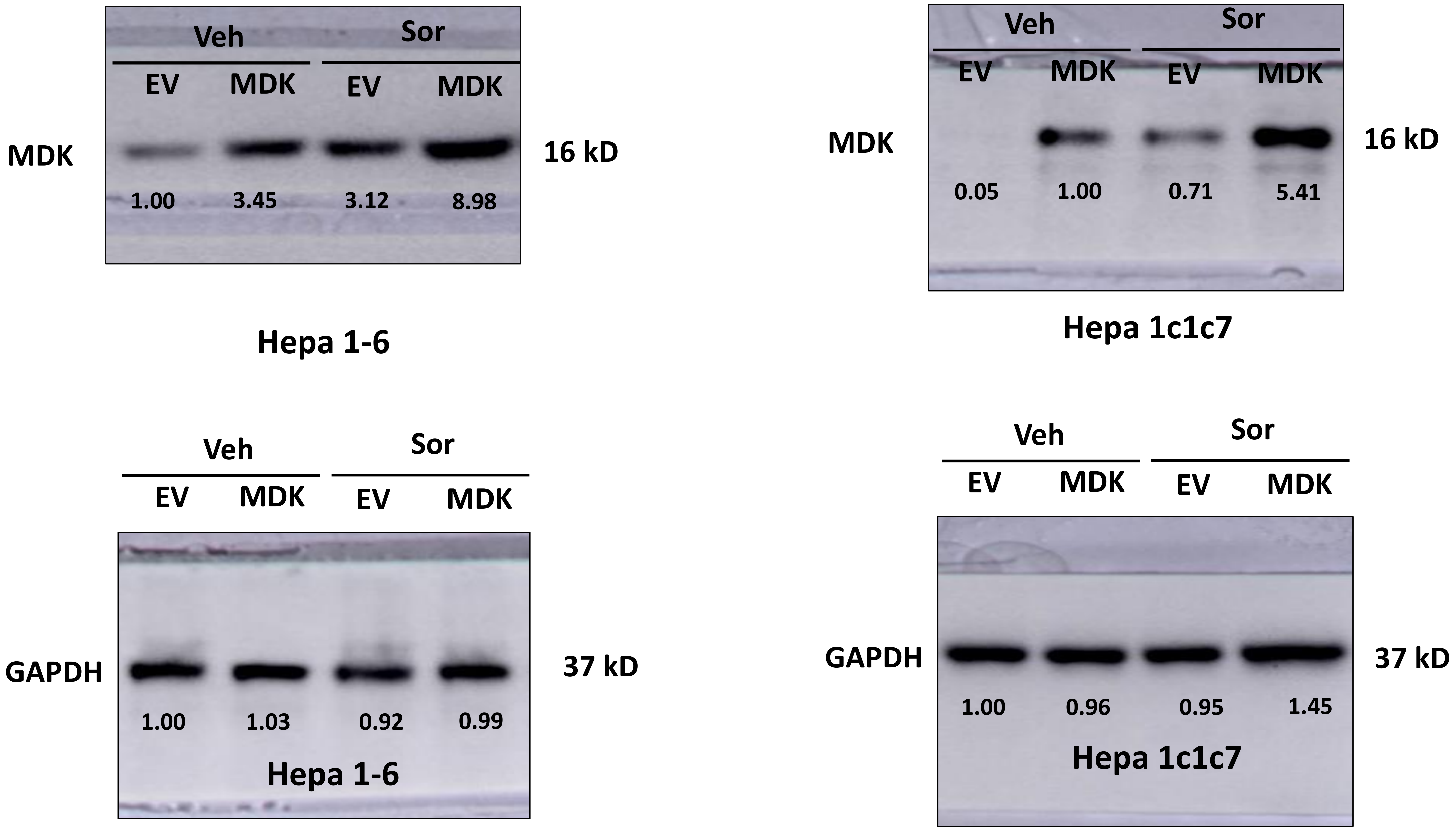

Veh
Sor
EV
MDK
EV
MDK
MDK
16 kD
1.00
3.45
3.12
8.98
Hepa 1-6
Veh
Sor
EV
MDK
EV
MDK
MDK
16 kD
0.05
1.00
0.71
5.41
Hepa 1c1c7
Veh
Sor
EV
MDK
EV
MDK
GAPDH
37 kD
1.00
1.03
0.92
0.99
Hepa 1-6
Veh
Sor
EV
MDK
EV
MDK
GAPDH
37 kD
1.00
0.96
0.95
1.45
Hepa 1c1c7

Fig 5A

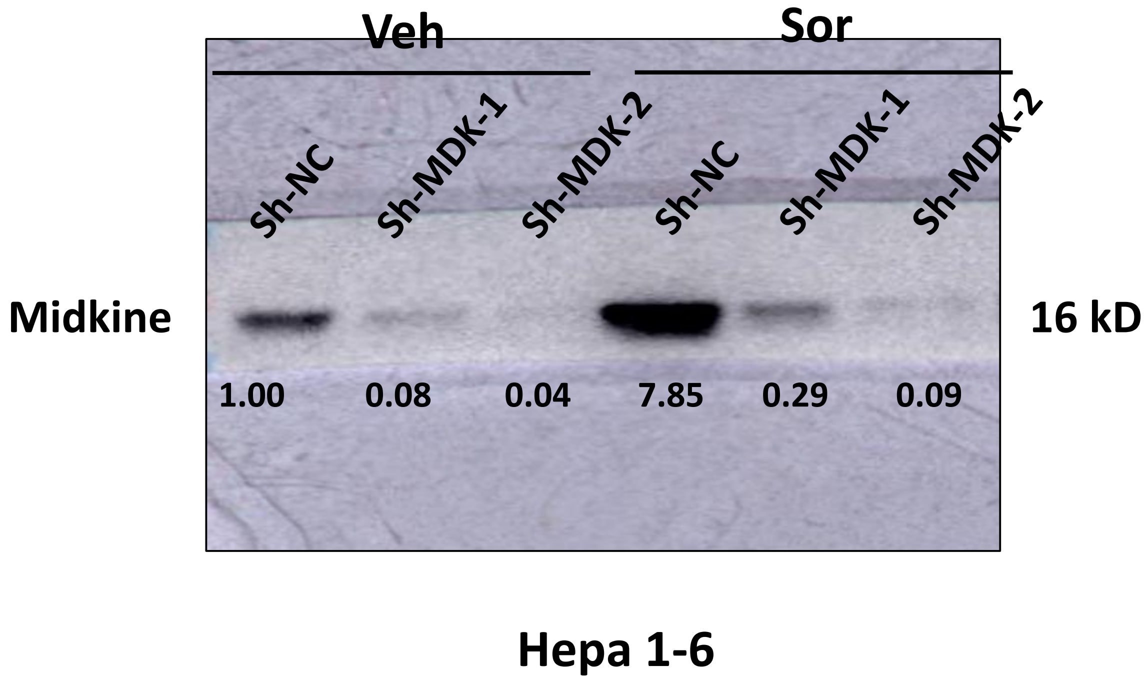
Veh
Sor
Sh-NC
Sh-MDK-1
Sh-MDK-2
Sh-NC
Sh-MDK-1
Sh-MDK-2
Midkine
16 kD
1.00
0.08
0.04
7.85
0.29
0.09
Hepa 1-6

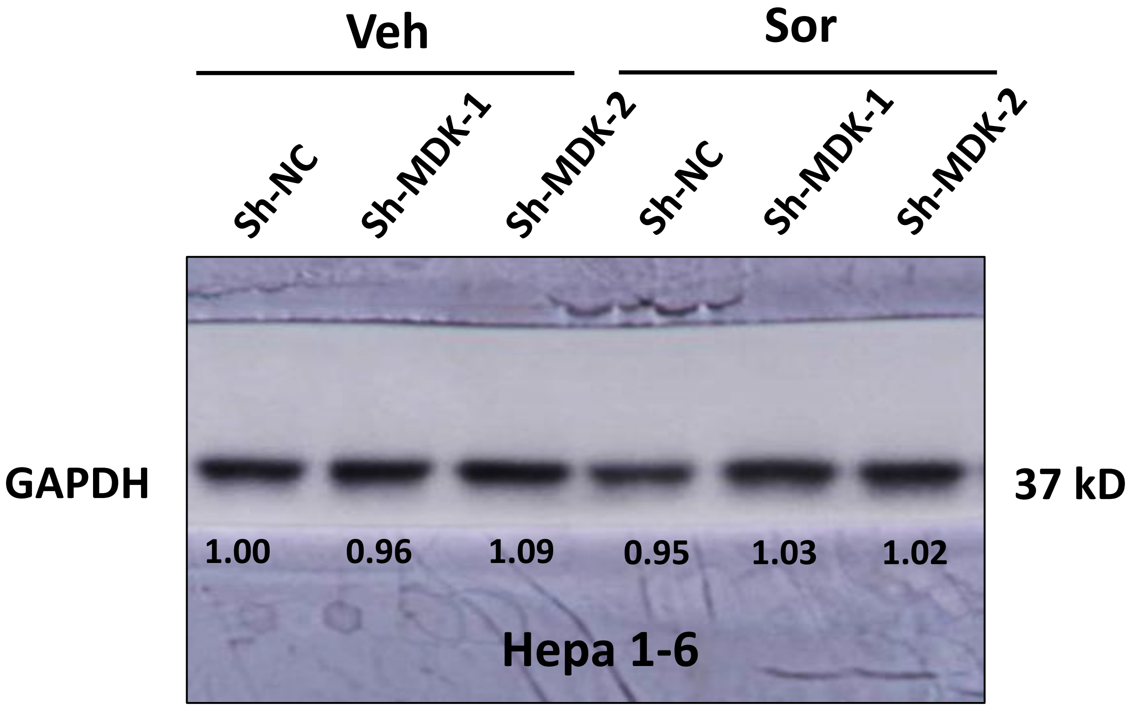
Veh
Sor
Sh-NC
Sh-MDK-1
Sh-MDK-2
Sh-NC
Sh-MDK-1
Sh-MDK-2
GAPDH
37 kD
1.00
0.96
1.09
0.95
1.03
1.02
Hepa 1-6

Fig 7A

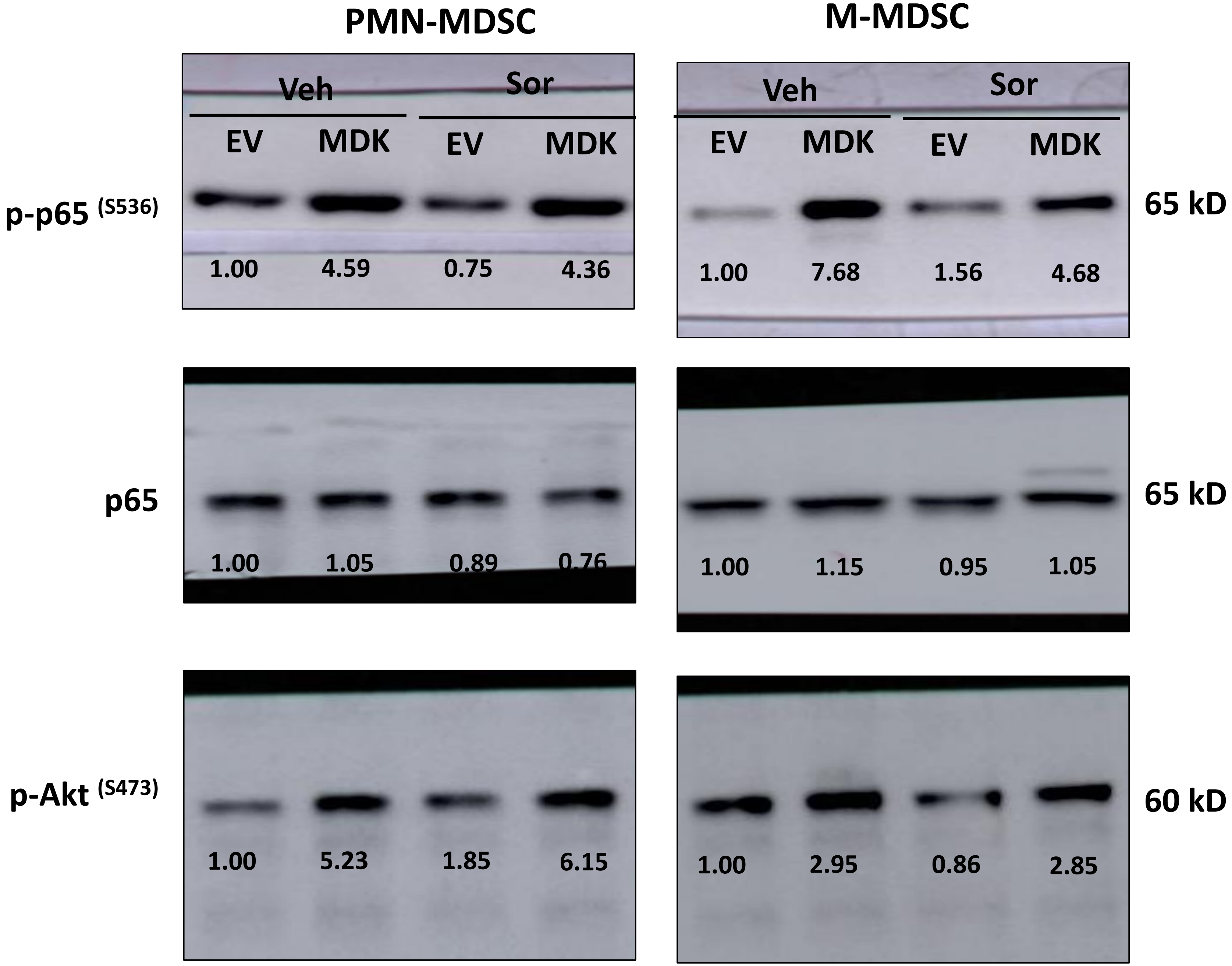

PMN-MDSC
M-MDSC
Veh
Sor
EV
MDK
EV
MDK
p-p65 (S536)
1.00
4.59
0.75
4.36
1.00
7.68
1.56
4.68
65 kD
p65
1.00
1.05
0.89
0.76
1.00
1.15
0.95
1.05
65 kD
p-Akt (S473)
1.00
5.23
1.85
6.15
1.00
2.95
0.86
2.85
60 kD

Fig 7A

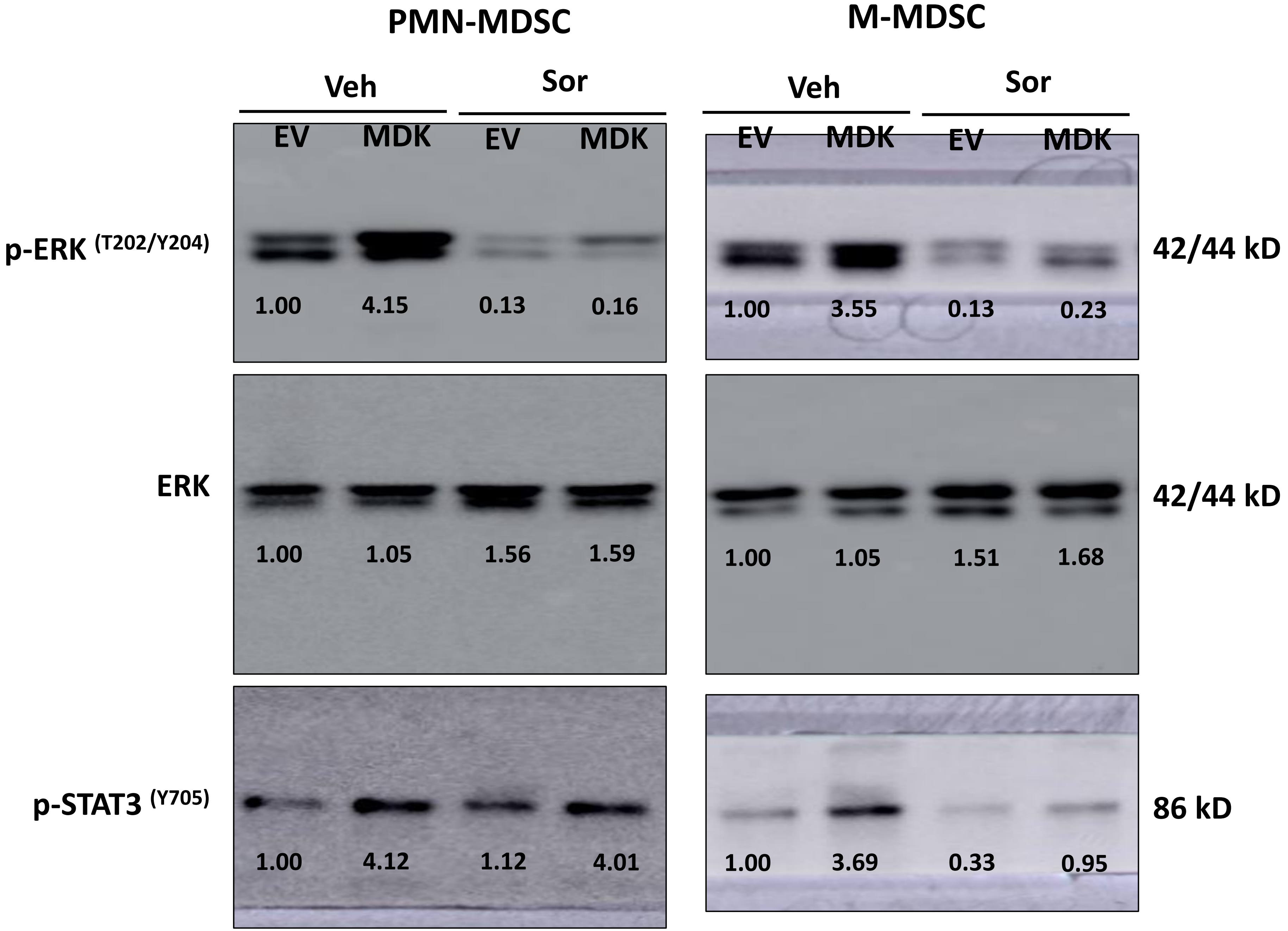

PMN-MDSC
M-MDSC
Veh
Sor
Veh
Sor
EV
MDK
EV
MDK
EV
MDK
EV
MDK
p-ERK (T202/Y204)
1.00
4.15
0.13
0.16
1.00
3.55
0.13
0.23
42/44 kD
ERK
1.00
1.05
1.56
1.59
1.00
1.05
1.51
1.68
42/44 kD
p-STAT3 (Y705)
1.00
4.12
1.12
4.01
1.00
3.69
0.33
0.95
86 kD

Fig 7A

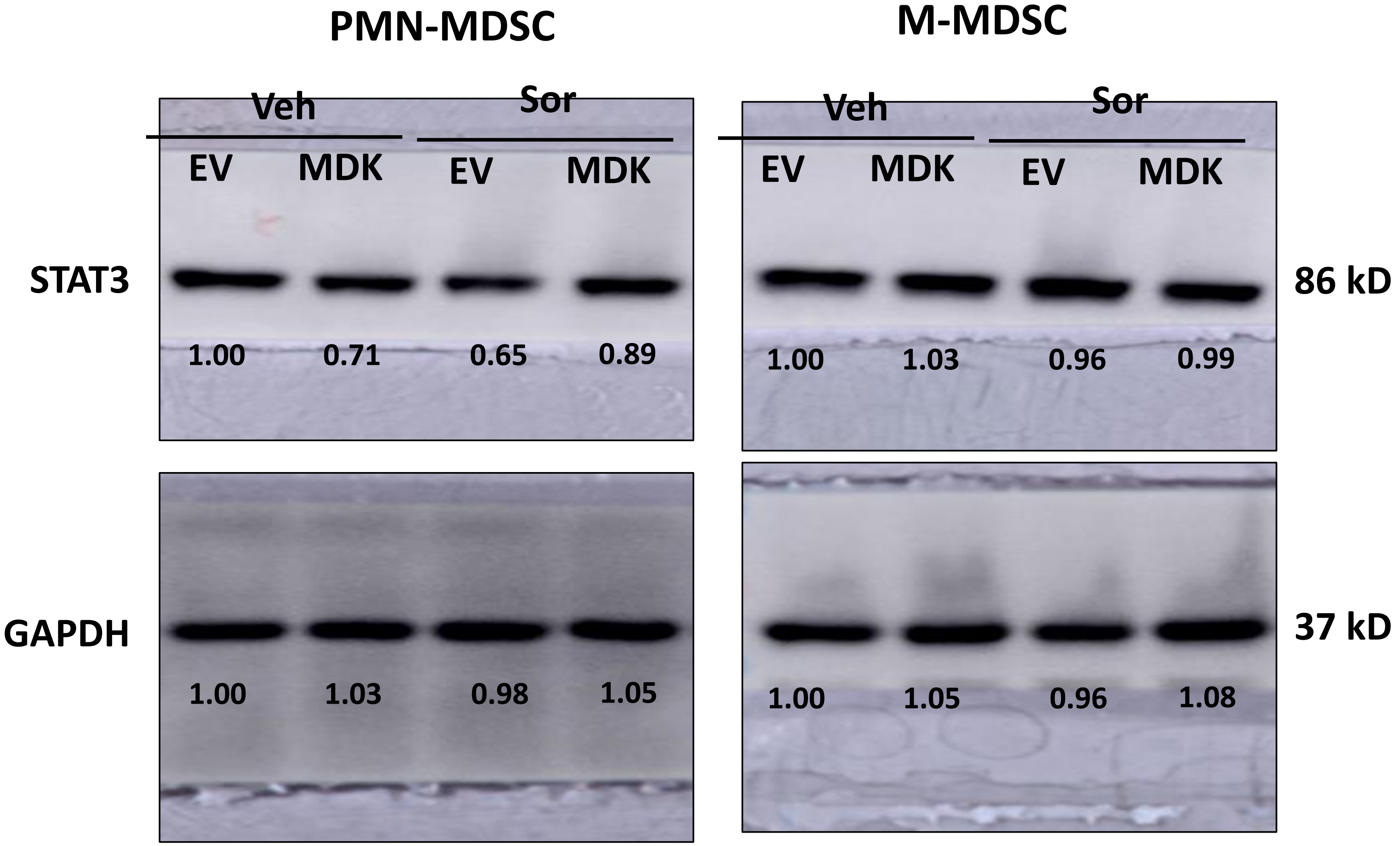
PMN-MDSC
M-MDSC
Veh
Sor
EV
MDK
EV
MDK
Veh
Sor
EV
MDK
EV
MDK
STAT3
1.00
0.71
0.65
0.89
1.00
1.03
0.96
0.99
86 kD
GAPDH
1.00
1.03
0.98
1.05
1.00
1.05
0.96
1.08
37 kD

Figure S1A

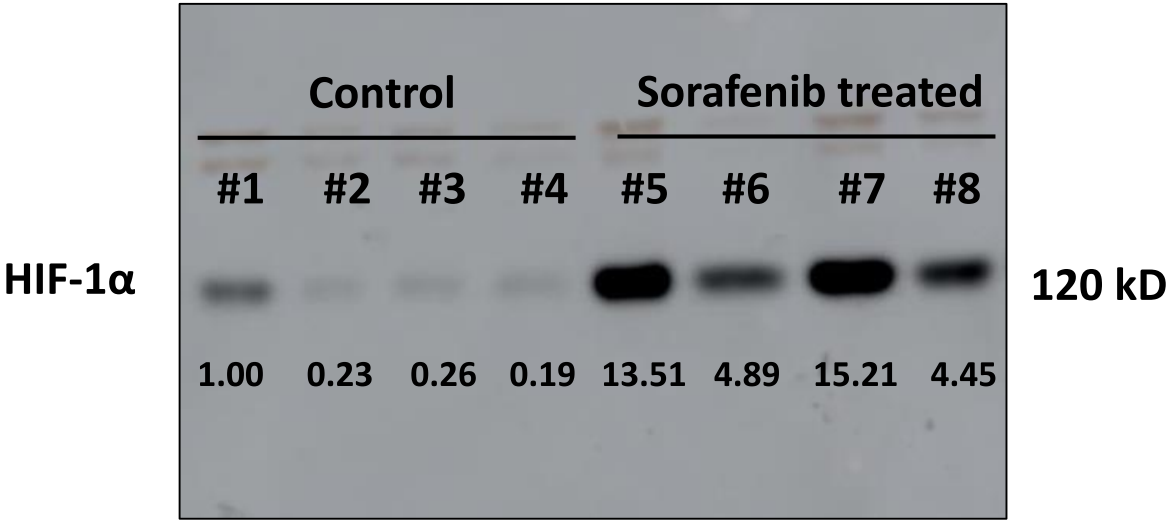
HIF-1α
Control
Sorafenib treated
#1 #2 #3 #4 #5 #6 #7 #8
1.00 0.23 0.26 0.19 13.51 4.89 15.21 4.45
120 kD

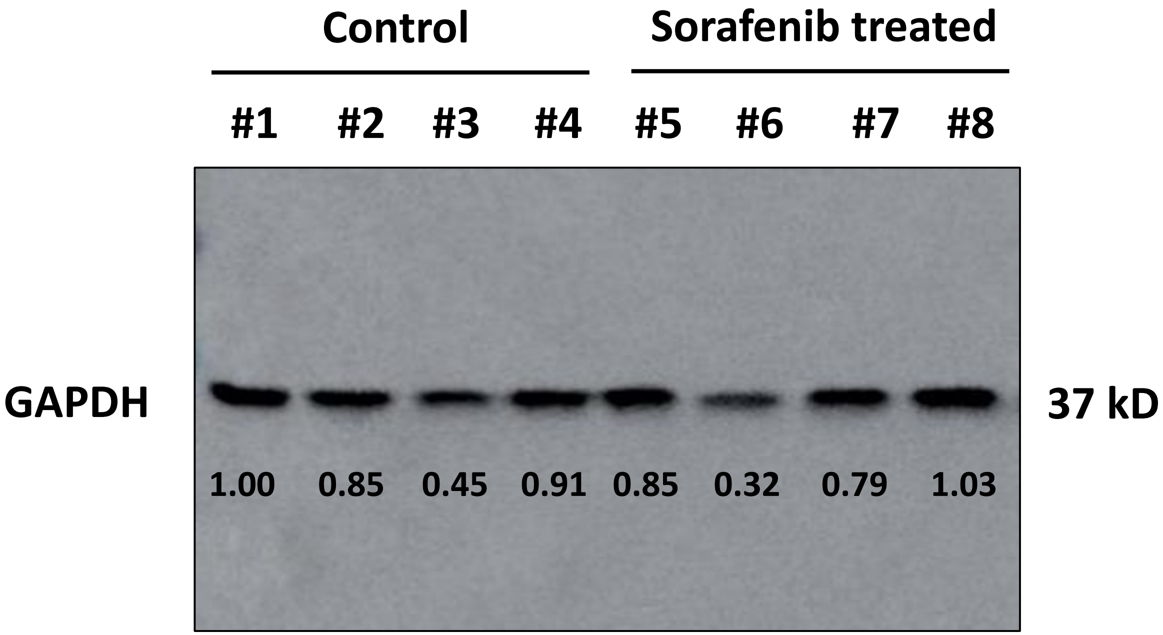
Control
Sorafenib treated
#1 #2 #3 #4 #5 #6 #7 #8
GAPDH
1.00 0.85 0.45 0.91 0.85 0.32 0.79 1.03
37 kD

Fig S6A

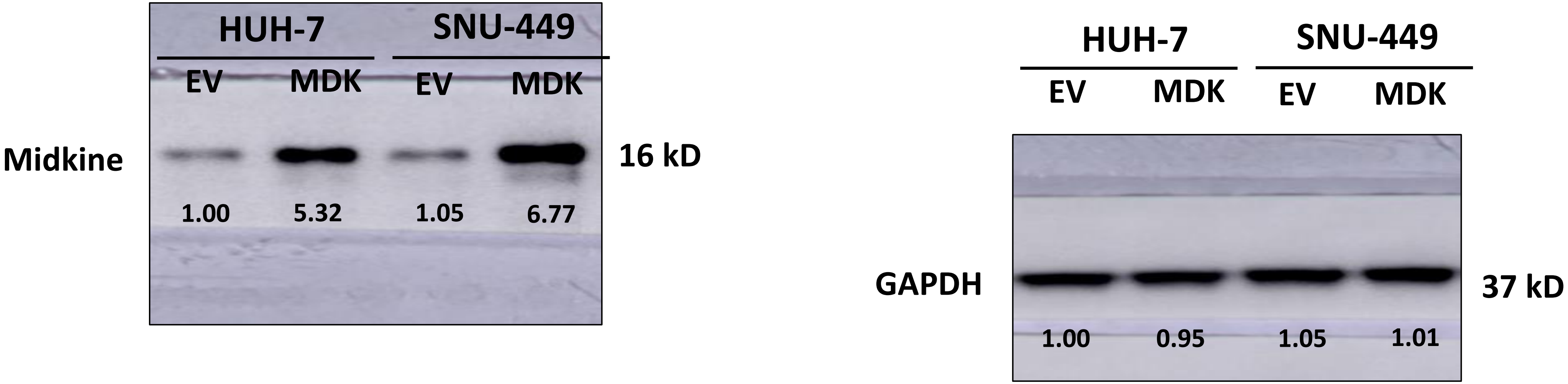
HUH-7
SNU-449
EV
MDK
EV
MDK
Midkine
16 kD
1.00
5.32
1.05
6.77
HUH-7
SNU-449
EV
MDK
EV
MDK
GAPDH
37 kD
1.00
0.95
1.05
1.01

Fig S7A

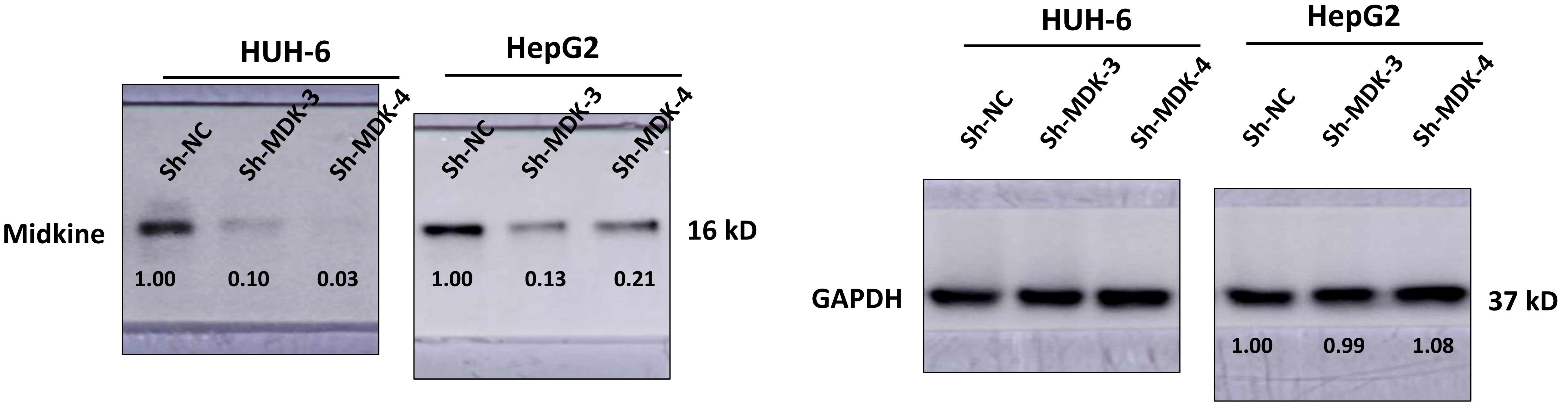
HUH-6
HepG2
Sh-NC
Sh-MDK-3
Sh-MDK-4
Midkine
16 kD
1.00
0.10
0.03
1.00
0.13
0.21
GAPDH
37 kD
1.00
0.99
1.08

Fig S8A

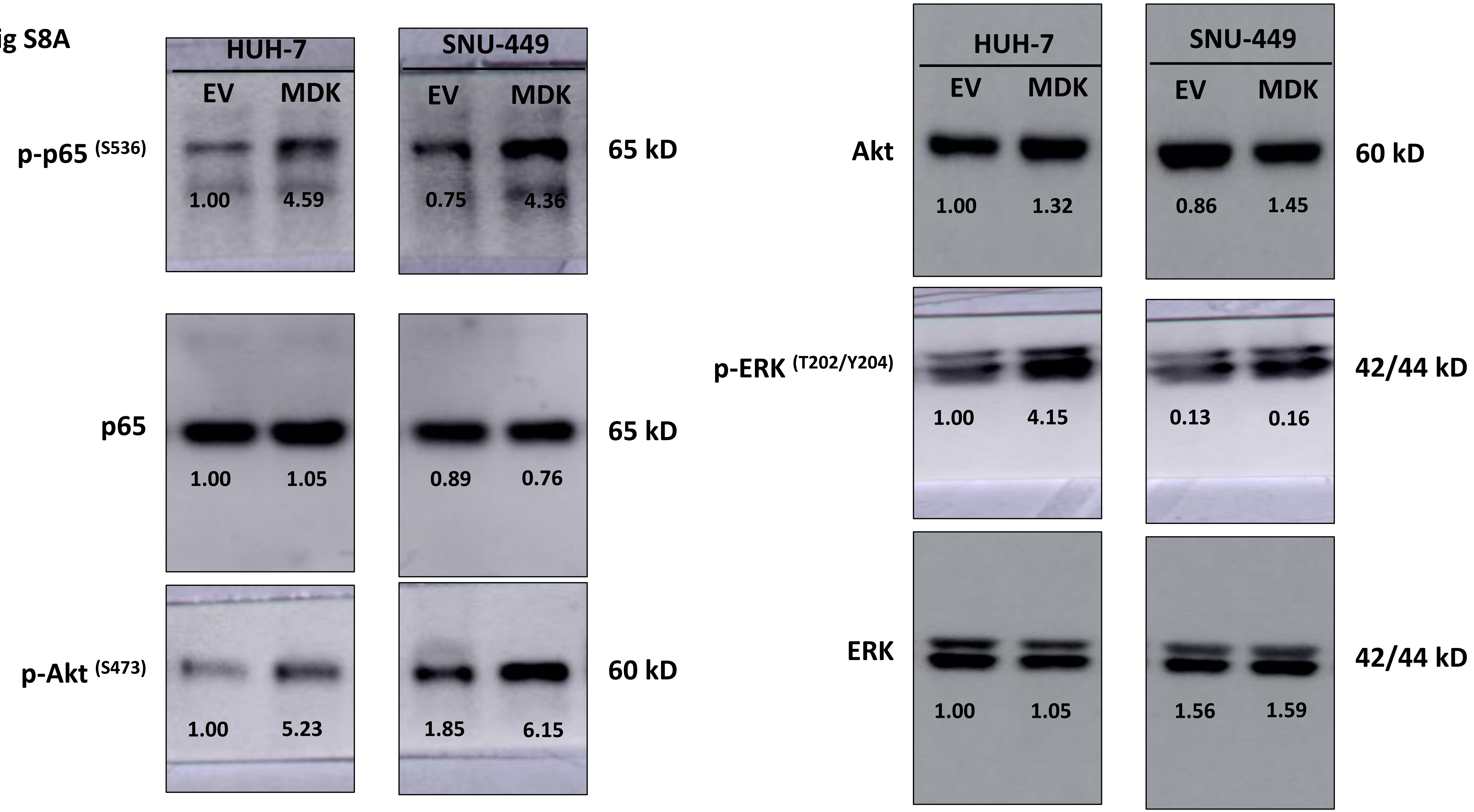
HUH-7
SNU-449
EV
MDK
EV
MDK
p-p65 (S536)
1.00
4.59
0.75
4.36
65 kD
p65
1.00
1.05
0.89
0.76
65 kD
p-Akt (S473)
1.00
5.23
1.85
6.15
60 kD
HUH-7
SNU-449
EV
MDK
EV
MDK
Akt
1.00
1.32
0.86
1.45
60 kD
p-ERK (T202/Y204)
1.00
4.15
0.13
0.16
42/44 kD
ERK
1.00
1.05
1.56
1.59
42/44 kD

Fig S8A

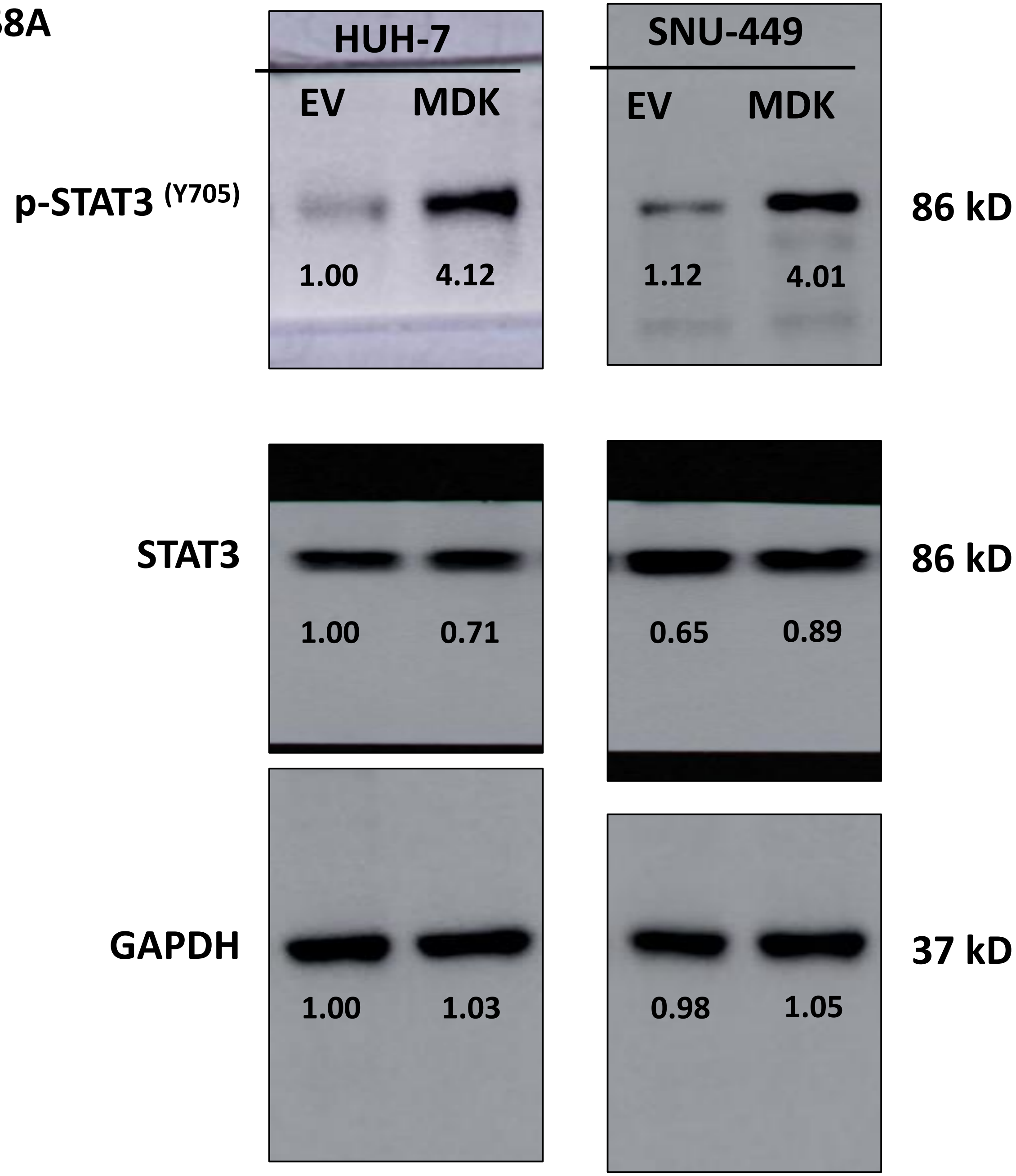
HUH-7
SNU-449
EV
MDK
EV
MDK
p-STAT3 (Y705)
1.00
4.12
1.12
4.01
86 kD
STAT3
1.00
0.71
0.65
0.89
86 kD
GAPDH
1.00
1.03
0.98
1.05
37 kD
